# Overexpression of SbSI-1, A Nuclear Protein from *Salicornia brachiata* Confers Drought and Salt Stress Tolerance and Maintains Photosynthetic Efficiency in Transgenic Tobacco

**DOI:** 10.3389/fpls.2017.01215

**Published:** 2017-07-13

**Authors:** Jyoti Kumari, Pushpika Udawat, Ashish K. Dubey, Md Intesaful Haque, Mangal S. Rathore, Bhavanath Jha

**Affiliations:** ^1^Marine Biotechnology and Ecology Division, CSIR-Central Salt and Marine Chemicals Research Institute, Council of Scientific and Industrial Research Bhavnagar, India; ^2^Academy of Scientific and Innovative Research, Council of Scientific and Industrial Research New Delhi, India

**Keywords:** abiotic stress, drought, halophyte, oxidative damage, salt, and transgenic

## Abstract

A novel S*alicornia* b*rachiata* Salt Inducible (*SbSI*-1) gene was isolated and overexpressed in tobacco for *in planta* functional validation subjected to drought and salt stress. *SbSI*-1 is a nuclear protein. The transgenic tobacco overexpressing *SbSI*-1 gene exhibited better seed germination, growth performances, pigment contents, cell viability, starch accumulation, and tolerance index under drought and salt stress. Overexpression of *SbSI*-1 gene alleviated the build-up of reactive oxygen species (ROS) and curtailed the ROS-induced oxidative damages thus improved the physiological health of transgenic tobacco under stressed conditions. The higher activities of antioxidant enzymes, lower accumulation of ROS, higher membrane stability, relative water content, and polyphenol contents indicated the better survival of the transgenic tobacco than wild-type (WT) tobacco under stressed conditions. Transgenic tobacco had a higher net photosynthetic rate, PSII operating efficiency, and performance index under drought and salt stress. Higher accumulation of compatible solutes and K^+^/Na^+^ ratio in transgenic tobacco than WT showed the better osmotic and redox homeostasis under stressed conditions. The up-regulation of genes encoding antioxidant enzymes (*NtSOD, NtAPX*, and *NtCAT*) and transcription factors (*NtDREB2* and *NtAP2*) in transgenic tobacco under stressed conditions showed the role of *SbSI*-1 in ROS alleviation and involvement of this gene in abiotic stress tolerance. Multivariate data analysis exhibited statistical distinction among growth responses, physiological health, osmotic adjustment, and photosynthetic responses of WT and transgenic tobacco under stressed conditions. The overexpression of *SbSI*-1 gene curtailed the ROS-induced oxidative damages and maintained the osmotic homeostasis under stress conditions thus improved physiological health and photosynthetic efficiencies of the transgenic tobacco overexpressing *SbSI*-1 gene.

## Introduction

The environmental stresses like salinity, drought, extreme temperature/light, water logging, mineral deficiencies, and pathogen attack etc. impinge on plant growth and development. Increasing salinization of soil and water as well as the scarcity of water resources (i.e., salinity and drought stress) are important factors representing primary cause of crop-productivity-loss globally (Vinocur and Altman, 2005). The detrimental effects of salinity stress on plant growth include metabolic imbalance, membrane damage, excess accumulation of reactive oxygen species (ROS), reduction in photosynthetic performance and depressing the nutrient uptake (Hasegawa et al., 2000). The environmental stresses are multigenic in nature and the biochemical pathways leading stress-induced cellular responses are interconnected. The stress tolerance/responses in a plant result from re-adjustment at physio-biochemical and molecular level through the coordinated action of several hundreds of stress responsive proteins, which enable plants to perform or survive better under stressed environment.

The demand to feed the growing world population with limited water and land resources necessities the need of crop breeding programs for development of salt and drought tolerant plants. Plants exhibit significant genetic diversity for stress tolerance (Ramegowda et al., 2012) and halophytes are the unique group of plants which tolerate excess salinity in the surroundings. The salt tolerance in halophytes is a multi-gene governed complex trait (Katschnig et al., 2015). Halophytes employ combinations of different strategies to handle excess salt in the surrounding. The molecular mechanism of stress responses plays a key role in devising strategies for development of salt tolerance in plants. Further understanding of the molecular mechanism of abiotic stress tolerance is continuously growing (Tuteja et al., 2013; Katschnig et al., 2015; Shukla et al., 2015; Nath et al., 2016; Srivastava et al., 2016; Udawat et al., 2016). With available knowledge of the molecular regulatory mechanism of salt tolerance, the choice of an appropriate transgene plays a critical role in devising strategy to develop salt tolerance in plants. The knowledge generated by elucidation of regulatory molecular mechanism would help to devise novel strategies to obtain more resilient plants with improved stress tolerance relevant to food production and environmental sustainability.

Genes conferring salt tolerance limit salt uptake from surroundings and transport throughout plants, adjust redox and osmotic balance, and regulate plant growth and development. The stress responsive genes in plants are categorized as functional and regulatory genes. The expressed sequence tags (ESTs) analysis is a powerful tool to elucidate differential gene expression and provides an opportunity to identify novel/unknown genes thus generating a candidate pool to find novel genes for stress tolerance. EST databases have been generated in glycophytes and halophytes in response to different environmental stresses (Wang et al., 2004; Amtmann et al., 2005; Vinocur and Altman, 2005; Reddy et al., 2008; Jha et al., 2009). EST database of halophytes show a large number of unknown genes and it can be theorized that unknown genes constitute a unique genetic makeup which helps plant to cope up with different environmental stresses (Yadav et al., 2014). *Salicornia brachiata* (Roxb.) is an extreme eu-halophyte and requires salt for its optimum growth (Katschnig et al., 2015). It can serve as a model for understanding salt adaptation/tolerance mechanisms (Lv et al., 2012; Rathore et al., 2015) thus facilitating the development of salt tolerance in crop plants. Being a potential resource of salt responsive genes, EST database was generated in response to salt stress (Jha et al., 2009). The EST database of *S. brachiata* contains a large number of novel/unknown/hypothetical genes (Jha et al., 2009) which might be playing a key role in salt tolerance mechanism in *S. brachiata*. The stress tolerance potential of the plant along with a large number of unknown genes makes this plant as a valuable genetic resource for engineering the abiotic stress tolerance in crop plants. Various salt responsive genes *viz. SbSI-2* (Yadav et al., 2014), *SbMYB15* (Shukla et al., 2015), *SbpAPX* and *SbGSTU* (Tiwari et al., 2013, 2016), *SbUSP* (Udawat et al., 2016), *SbSLSP*, and *SbSDR*1 (Singh et al., 2016a,b) have been isolated from *S. brachiata* and characterized. The *SbSI*-1 (Salt-Induced gene 1 from S. b*rachiata*) and *SbSI*-2 genes were cloned full-length and studied *in silico*. The *SbSI*-1 was expressed in *E. coli* (Yadav et al., 2012) and *SbSI*-2 in tobacco (Yadav et al., 2014). The *SbSI*-1 (JF965339.1) with an ORF of 480 bp encoded an 18.39 KDa protein of 159 amino acids with pI 8.58 while *SbSI*-2 (JX872273.1) with an ORF of 423 bp encoded an 15.93 KDa protein of 140 amino acids with pI 10.34. The *SbSI*-1 and *SbSI*-2 differed 55.24 and 75.86% at nucleotide and polypeptide level, respectively. The heterologous expression of *SbSI*-1 conferred to drought and salinity tolerance and revealed it as a potential candidate gene to develop drought and salt tolerance in plants. In the present study, *SbSI*-1 gene was transformed into tobacco (*Nicotiana tabacum* cv. petit havana). Biochemical, physiological and morphological responses of transgenic tobacco overexpressing *SbSI*-1 gene under control of CaMV 35S promoter was studied under drought (10% PEG) and salt (200 mM NaCl) stress.

## Materials and methods

### Subcellular localization of SbSI-1 protein

SbSI-1 protein localization was examined *in vivo* using Gateway technology (Walhout et al., 2000). RNA was isolated from *S. brachiata* and cDNA was synthesized using a SuperScript RT III first-strand cDNA synthesis kit. The *SbSI*-1 CDS was amplified with AccuPrime™ Pfx DNA polymerase using *SbSI*-1LF and *SbSI*-1LR primers. Subsequently, the amplified CDS was cloned into a pENTER/D-TOPO entry vector (Invitrogen, USA). LR recombination reaction was performed between an attL-containing Entry clone pENTER/D-TOPO-*SbSI*-1 vector and an attR-containing destination vector pSITE-4CA using Gateway LR Clonase II enzyme mix (Invitrogen, USA). Integration of *SbSI*-1 gene in destination vector was confirmed by sequencing. The control vector and expression cassette (RFP:*SbSI*-1) (Supplementary Figures 1A,B) were transformed into onion epidermal cells using gene gun (PDS-1000/He Biolistic, Biorad, USA). The transformed epidermal cells were observed for transient expression of RFP with an epifluorescence microscope (Axio Imager, Carl Zeiss AG, Germany) after an incubation of 24 h on Murashige and Skoog's (MS) basal medium (Murashige and Skoog, 1962). Further the electrophoretic mobility shift assay (EMSA) of SbSI-1 protein was performed following Uguru et al. (2005) to check DNA binding property. The DNA samples was incubated with recombinant protein. After incubation the sample were electrophoresed in 6% native PAGE. The gel was silver stained and documented to record electrophoretic mobility shift.

### Construction of plant expression vector and transformation of tobacco

The *SbSI*-1 gene was amplified using *S. brachiata* cDNA with AccuPrime™ Pfx DNA polymerase using forward (5′-TCCGAGCTCATGCCTAATAAACATATCATGG-3′) and reverse (5′-CGCGGATCCTTAACGGTTCCCTTGTTTC-3′) primers containing *Sac* I and *BamH* 1 sites, respectively. The *SbSI*-1 amplicon with *BamH* 1/*Sac* I sites was cloned into pRT101 vector (Töpfer et al., 1987). Further, the cassette containing the CaMV35S constitutive promoter, *SbSI*-1 gene and terminator was cloned into the pCAMBIA2301 vector at *Pst* I site (Supplementary Figure 1C) and subsequently mobilized into *Agrobacterium tumefaciens* (LBA 4404). Tobacco (*Nicotiana tabacum* cv. petit havana) was transformed following the standard protocol (Horsch et al., 1985). Putative transgenic shoots regenerated in the presence of 50 mgl^−1^ kanamycin were elongated, rooted, acclimatized and grown under containment facility to harvest the T_0_ seeds.

### Molecular analysis of transgenic tobacco

The putative transgenic (T_0_ and T_1_) lines were checked for transformation through histochemical GUS assay (Jefferson, 1987) using β-glucuronidase reporter gene staining kit (Sigma, USA) and PCR amplification of *uid*A and *SbSI*-1 gene (Supplementary Table 1). The number of homologs of *SbSI*-1 gene in *S. brachiata* and the transgene integration in transgenic tobacco was confirmed through southern blot analysis. To determine the homologs of *SbSI*-1, 30 μg genomic DNA from *S. brachiata* was digested with *Eco*R 1 and *Xho* 1 restriction enzymes. To determine the transgene integration 20 μg genomic DNA from WT and transgenic tobacco were digested with *Hin*d III. The digests were separated on 0.8% agarose gel by electrophoresis. Subsequently these were transferred onto the Hybond N^+^ membrane (Amersham Pharmacia, UK) using buffer (0.4 N NaOH with 1 M NaCl) to generate DNA blot. The blot was hybridized with PCR-generated probe for the *SbSI*-1 gene labeled with DIG-11-dUTP. Pre-hybridization and hybridization were carried out at 42°C overnight in DIG EasyHyb buffer solution (Roche, Germany). The hybridized products were detected using CDP-Star chemi-luminescent as substrate, following the manufacturer user guidelines (Roche, Germany) and signals were visualized and documented on X-ray film after 30 min. Transgene expression in transgenic tobacco was studied through semi-quantitative reverse transcriptase (RT) PCR analysis using *actin* as an internal control.

To study the involvement of *SbSI*-1 gene in abiotic stress tolerance, qRT PCR (Bio-Rad IQ5, Bio-Rad, USA) was performed in WT and transgenic tobacco under different (10% PEG, 200 mM NaCl, 20 μM ABA and 10 μM SA) stresses using QuantiFast Kit (Qiagen, USA). The expression pattern of *NtSOD, NtCAT*, and *NtAPX* genes encoding different antioxidant enzymes (Huang et al., 2011) and *NtDREB*2 and *NtAP*2 encoding transcription factors were analyzed (Supplementary Table 1). The qRT-PCR specificity was monitored by melt curve analysis and fold expression rate was calculated by CT method (Livak and Schmittgen, 2001).

### Analysis of *SbSI*-1 transgenic plants under different abiotic stresses

Seeds of wild-type (WT) plant and transgenic (L_8_, L_22_, and L_33_) lines overexpressing *SbSI*-1 were germinated on MS medium supplemented with 200 mM mannitol or 200 mM NaCl (20 seeds per replicate). The germination was carried out under a culture room conditions [26 ± 2°C, 55–60% relative humidity (RH), 12 h photoperiod with a light intensity of 35 ± 5 μmol m^−2^ s^−1^ SFP]. Seed germination was recorded after 21 days (d) and germination percent was calculated. Uniform WT and kanamycin-positive T_1_ transgenic seedlings were cultured vertically onto MS medium containing 200 mM mannitol or 200 mM NaCl for 21 d. The different growth parameters (plant height, shoot/root length, fresh and dry weight) and ion contents (Na^+^ and K^+^) were measured in these plants and compared with WT.

The seeds were germinated on kanamycin-supplemented medium for functional characterization. Uniform WT and kanamycin-positive T_1_ transgenic seedlings were cultured hydroponically in the ½ strength of Hogland salt for 45 d under culture room conditions. These were subjected to 10% polyethylene glycol (PEG) and 200 mM NaCl for 24 h; thereafter samples were harvested for morphological (growth parameters) and physio-biochemical (relative water contents, electrolyte leakage, membrane stability index, tolerance index, H_2_O_2_ estimation, lipid peroxidation, ROS, and starch accumulation, osmotic adjustment, estimation of antioxidant enzyme activity and determination of sugar, polyphenols, and free amino acid contents) analysis in T_1_ transgenic tobacco along with WT.

### Leaf senescence assay, photosynthetic pigment estimation, and cell viability assay

Uniform leaf disks (5–6 mm diameter) punched from leaf lamina of 45 d old WT and transgenic lines were used for the leaf senescence assay, chlorophyll estimation, and cell viability assay. Leaf disks (*n* = 11) of WT and transgenic lines were floated in 5 ml ½ strength of Hoagland salt (control) supplemented with 100, 150, 200 mM NaCl, and 10% PEG for 7 d. The retention of greenish color by leaf disks was recorded as phenotypic effects of stress treatments. Leaf disks at the end of stress period were used for photosynthetic pigment estimation (Inskeep and Bloom, 1985; Chamovitz et al., 1993) and 2,3,5-triphenyltetrazolium chloride (TTC) assay for cell viability estimation (Towill and Mazur, 1975; Hema et al., 2014).

### Gas exchange and chlorophyll fluorescence

Simultaneous gas exchange and chlorophyll fluorescence parameters were measured in leaves of control and 24 h stress treated WT and transgenic tobacco using LI-6400XT portable photosynthesis system (LICOR, Lincoln, NE, USA). Plants were dark-adapted for 45 min and fluorescence parameters were recorded. Steady state fluorescence yield was achieved by exposing plants to ambient light (1,200 μmol m^−2^s^−1^) for 60 min in a plant growth chamber (PGC-105, Percival Scientific, US). The gas exchange measurement conditions were 1,200 μmol m^−2^ s^−1^ PPFD, ambient atmospheric CO_2_ (380 μmol^−1^ mol^−1^) and RH (60–65%) and 26°C block temperature. The net photosynthetic rate (P_N_; μmol m^−2^s^−1^), stomatal conductance (g_s_; mmol H_2_O m^−2^s^−1^), intercellular CO_2_ (C_i_) concentration (μmol m^−2^s^−1^), transpiration rate (E; mmol m^−2^s^−1^), PSII operating efficiency (ΦPSII), electron transport rate (ETR), photochemical quenching (qP), non-photochemical quenching (NPQ) were determined.

Chlorophyll a fluorescence transient (Strasser and Strasser, 1995) in leaves was measured on dark adapted stressed and unstressed plant using plant efficiency analyser (Hansatech Instruments Ltd., England). Chlorophyll fluorescence was recorded thrice (sub-replicate) at different regions of 03rd leaf of 03 plants (biological replicate) from each treatment (*n* = 9).

### Relative water content, electrolyte leakage, membrane stability, and tolerance index

Leaf disks were punched from leaf lamina of stressed and unstressed plants and fresh weight (FW) was recorded. Subsequently turgid weight (TW) was recorded after submerging these disks in deionized water for 12 h. Dry weight (DW) was recorded after drying the disks at 80°C for 48 h in hot air oven. The relative water content (RWC) was calculated as: RWC (%) = (FW–DW/TW–DW) × 100.

The leaf samples from stressed and unstressed tobacco plants were washed with deionized water. These were immersed in 10 ml deionized water in a closed vial and incubated at 26°C on a gyratory shaker for 24 h. The electrical conductivity (EC) of the solution (EC_1_) was measured using conductivity meter (SevenEasy, Mettler Toledo AG 8603, Switzerland). These samples were autoclaved at 121°C for 15 min, cooled up to 26°C and EC (EC_2_) was measured. The electrolyte leakage (EL) was calculated as (EC_1_/EC_2_) × 100.

The uniform leaf disks from stressed and unstressed tobacco were immersed in 10 ml deionized water in close vials in two separate sets. A set of vials was incubated at 40°C for 30 min while the second set of vials at 100°C for 10 min. EC of both sets (EC_40_ for 40°C and EC_100_ for 100°C) was recorded and membrane stability index (MSI) was calculated (Sairam, 1994) as [1-(EC_40_/EC_100_)] × 100.

Uniform leaf samples from control and treated plants were dried and DW was recorded. The tolerance index (TI) was determined as DW of the plant under stress condition × 100/DW of the plant under control condition (Tuteja et al., 2013) and compared with that of WT plants.

### *In vivo* localization of H_2_O_2_, ${\text{O}}_{2}^{-}$, and starch

Hydrogen peroxide and ${\text{O}}_{2}^{-}$ radical accumulation were detected *in vivo* in leaves of stressed and unstressed tobacco. The leaves were immersed in nitro-blue tetrazolium (NBT) solution (1 mg mL^−1^ in 10 mM phosphate buffer; pH 7.8) at RT for 2 h, thereafter exposed to high irradiance for 12 h until blue spots showing accumulation of ${\text{O}}_{2}^{-}$ appeared. The presence of H_2_O_2_ was detected by immersing the leaf samples in 3,3-diaminobenzidine (DAB) solution (1 mg mL^−1^ in 10 mM phosphate buffer; pH 3.8) at RT for 6 h in the dark, thereafter exposing to the light until brown spots showing accumulation of H_2_O_2_ appeared. Before documentation, the samples were washed with ethanol to bleach out the chlorophyll. Uniform leaves from stressed and unstressed tobacco were processed for *in vivo* detection of starch following Cheng et al. (2014).

### Estimation of lipid peroxidation, antioxidant enzyme activity, H_2_O_2_, and accumulation of ${\text{O}}_{2}^{-}$ radicals

Lipid peroxidation was determined by estimating malondialdehyde (MDA) concentration following Hodges et al. (1999). Leaf samples were extracted with 0.1% trichloroacetic acid (TCA), and 0.2 ml extract was reacted with 0.8 ml of TBA reagent (0.5% TBA in 20% TCA) and subsequently boiled at 95°C for 30 min. Samples were ice cooled, centrifuged at 10,000 g for 5 min and absorbance was read at 440, 532, and 600 nm.

The leaf tissue from treated and untreated plants were ground in liquid nitrogen and extracted in protein extraction buffer (50 mM potassium buffer (pH 7.0), 1 mM EDTA, 0.05% (w/v) triton × −100, 5% (w/v) polyvinylpolypyrrolidone). The protein concentration in the extract was determined following Bradford method (Bradford, 1976) and it was used for determination of activity of superoxide dismutase (SOD), ascorbate peroxidase (APX), and catalase (CAT). The SOD activity was assayed by monitoring the inhibition of photochemical reduction of nitro blue tetrazolium (NBT) following Beyer and Fridovich (1987). The amount of enzyme required for 50% inhibition of NBT reduction as monitored at 560 nm was considered as one unit of SOD activity. The APX activity was assayed by monitoring oxidation of ascorbate (Nakano and Asada, 1981). The decline in absorbance at 290 nm was recorded and 2.8 mM^−1^ cm-1 was taken as extinction coefficient (Δe). The CAT activity was assayed by monitoring the disappearance of H_2_O_2_ (Miyagawa et al., 2000) and taking 43.6 M^−1^ cm^−1^ as Δe at 240 nm (Patterson et al., 1984).

To determine the H_2_O_2_ contents the leaf samples were extracted with 0.1% TCA. A 100 μl extract was reacted with 1 ml of reaction buffer containing 0.25 mM FeSO_4_, 0.25 mM (NH_4_)_2_SO_4_, 25 mM H_2_SO_4_, 1.25 mM xylenol orange and 1 mM sorbitol (He et al., 2000) at RT for 1 h and absorbance was read at 560 nm.

The accumulation of ${\text{O}}_{2}^{-}$ radicals were studied through XTT assay (Schopfer et al., 2001) by demonstrating the reduction of XXT into formazans (Able et al., 1998). Uniform leaf disks were incubated with 1 ml of XTT solution (500 mM XTT in 20 mM K-phosphate buffer, pH 6.0) in darkness at 25°C on a shaker. The increase in absorbance (A_470_) of the incubation medium was recorded (Sutherland and Learmonth, 1997).

### Osmotic adjustment and ion content analysis

The fresh leaf tissue from control and treated plants were frozen, thawed and centrifuged at 10,000 g to extract the sap. The solute concentration was determined using Vapro Pressure Osmometer (model-5600; Wescor, Logan UT, USA). The solute potential (Ψ_s_) of the sap was calculated as —nRT/V; where n represent a number of solute molecules; R is universal gas constant; T is the temperature in ° K, and V is volume in liter.

The treated and untreated WT and transgenic seedlings were oven dried, acid [perchloric acid and nitric acid solution (3:1 v/v)] digested and heated to dryness. The digest was dissolved in deionised water and ion contents (Na^+^ and K^+^) were estimated by inductively coupled plasma optical emission spectrometer (Optima2000DV, PerkinElmer, Germany).

### Quantification of proline, sugars, polyphenols, and free amino acid contents

The proline content in leaf tissues (200 mg) of stressed and unstressed tobacco were extracted with 3% sulphosalicylic acid and estimated following Ringel et al. (2003). The leaf tissues (1,000 mg) were extracted in 70% ethanol, and the residue left after evaporation was dissolved in milliQ water for estimation total soluble sugar, reducing sugar, polyphenol and free amino acid contents. The total soluble sugar and reducing sugar contents in leaves were measured following anthrone-sulphuric acid (Dubois et al., 1956) and DNS method (Miller, 1959), respectively. The polyphenol contents in leaves were determined following Chandler and Dodds (1983) using Folin-Ciocalteau's reagent. Total free amino acid contents in leaf tissues were determined following Sugano et al. (1975).

### Statistical analysis

Each experiment was performed three times with three biological replicates for physio-chemical estimations and with 10 replicates for growht measurments. The data recorded were subjected to one-way ANOVA for analysis of variance to determine the significance among mean values of WT and transgenic plants among treatments. LSD was used for comparisons of means. The data was presented as mean ± *SD* and significance in responses of transgenic lines against control was indicated by classical asterisks (^*^<0.05; ^**^<0.01; ^***^<0.001). The multivariate analysis (Principal Component Analysis; PCA) was carried out to study the correlation between different variables among multidimensional datasets. Plants grown under stresses and different parameters measured were considered as observations and variables, respectively. PCA was performed and analyzed using SigmaPlot (version 13) and Origin (version 15) for individual (for growth, physiological health, osmotic adjustment and photosynthetic parameters), and integrated (all parameters together) responses.

## Results

### The SbSI-1 protein is nuclear localized

**Figure 1:**
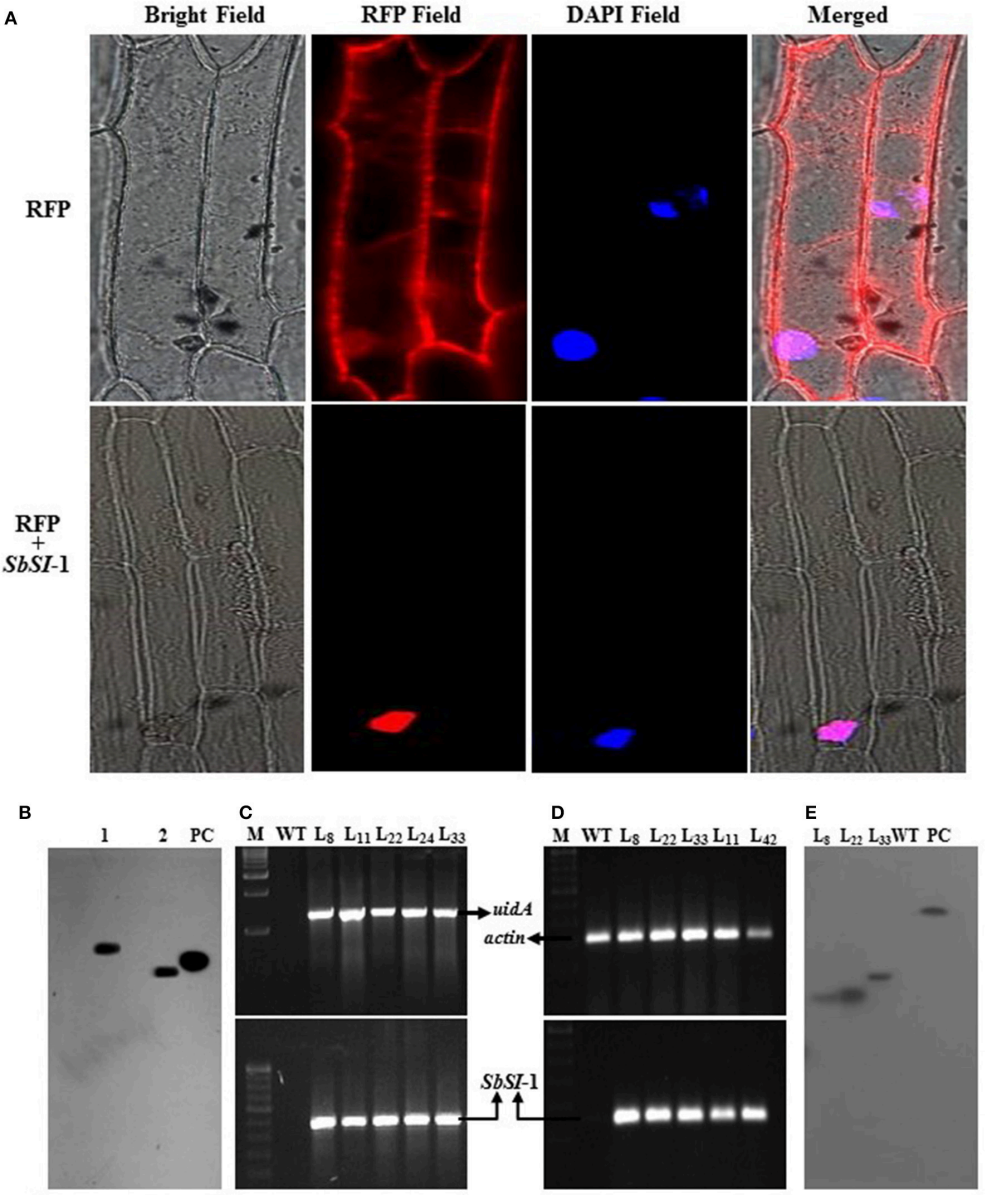
Subcellular localization of SbSI-1 protein, homolog determination and molecular analysis of transgenic tobacco. Transient expression of RFP alone and RFP:SbSI-1 translational fusion protein in onion epidermal cells **(A)**, determination of homologs of *SbSI*-1 in *S. brachiata* by southern blot analysis **(B)**, PCR confirmation of transgenic tobacco by *uid*A and *SbSI*-1 gene amplification **(C)**, semi-quantitative RT-PCR transgene-expression in transgenic tobacco using actin as an internal control **(D)**, and determination of transgene integration in tobacco by southern blot analysis **(E)**. The lane 1–2 are samples digested with *Eco*R 1 and *Xho* 1 respectively, PC is positive (*SbSI*-1 PCR product of ORF region) control, M is DNA ladder, WT-wild type plant/negative control and L_*X*_ are transgenic tobacco lines.

The transient expression assays of RFP and RFP:*SbSI*-1 fusion construct showed SbSI-1 protein as a nuclear-localized protein. Onion cells transformed with RFP alone exhibited an even distribution of red fluorescence signals in the entire cell region, whereas transformed cells with RFP:*SbSI*-1 fusion construct showed fluorescence signals in the nucleus only. DAPI stained (blue spot) nucleus showed overlapping with an RFP stained (red spot) nucleus (Figure 1A). In merged image fluorescence was observed only in the nucleus and not from any region in the cell confirming the nuclear localization of the SbSI-1 protein. The southern blot analysis confirmed single copy of *SbSI*-1 gene (Figure 1B) in *S. brachiata*. The EMSA further showed DNA binding property of SbSI-1 protein (Supplementary Figure 1D).

### Molecular confirmation of putative transgenic tobacco plants

At the end of transformation experiment 42 putative transgenic lines were obtained showing positive GUS assay (Supplementary Figures 1E, 2A). The transgenic tobacco plants (putative transgenic plants and T_1_ transgenic tobacco lines) were confirmed by GUS assay and PCR amplification of both *SbSI*-1 and *uid*A gene (Supplementary Figures 2A,B). Based on seed germination (data not shown), GUS expression assay and PCR analysis (Figure 1C) line L_8_, L_22_, and L_33_ were selected for functional validation. Semi-quantitative RT-PCR showed expression of *SbSI*-1 gene in transgenic tobacco whereas no expression was detected in WT plants (Supplementary Figure 2C; Figure 1D). The southern hybridization confirmed single copy gene integration to the selected transgenic lines (Figure 1E).

### *SbSI*-1 overexpression improved growth performance

**Figure 2:**
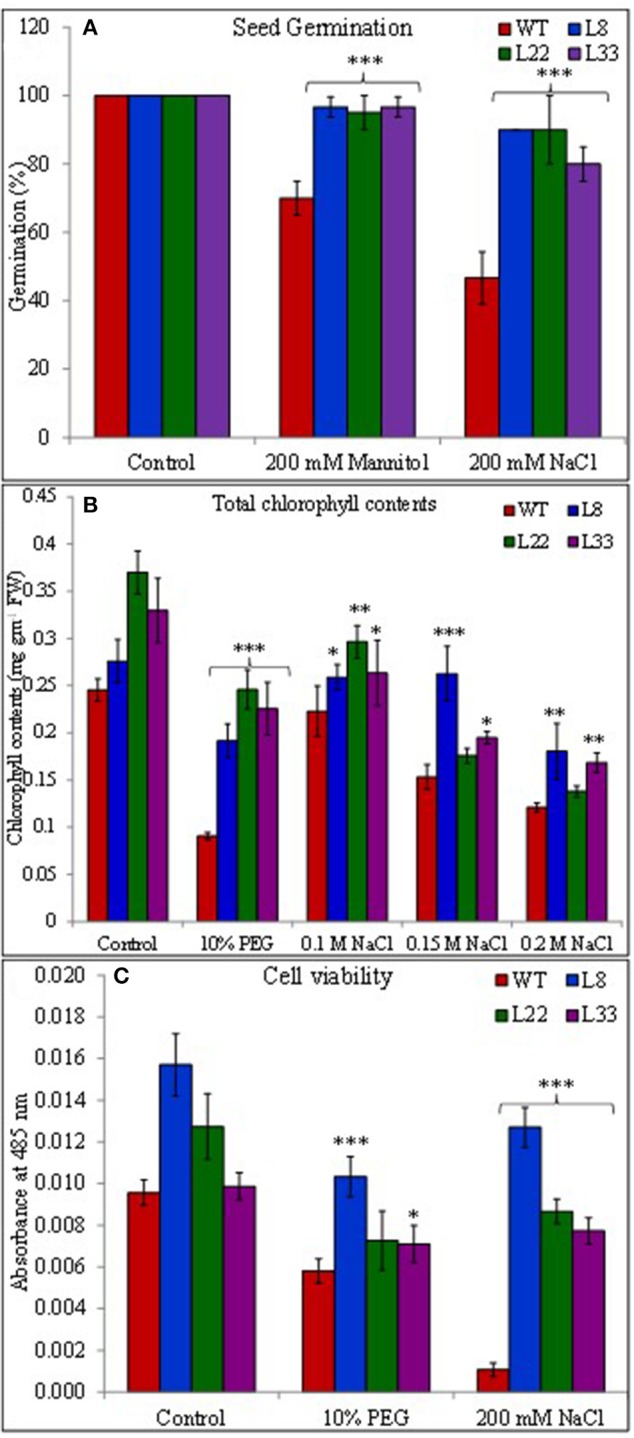
Seed germination in WT and transgenic lines under mannitol and NaCl stress after 21 days **(A)**, total chlorophyll contents **(B)**, and cell viability assay **(C)** of WT and transgenic tobacco under control and stress (PEG and NaCl) conditions for 7 days. The ^*^, ^**^, and ^***^ denote statistical significance in responses of transgenic lines against control at P value ≤0.05, 0.01, and 0.001 respectively.

**Figure 3:**
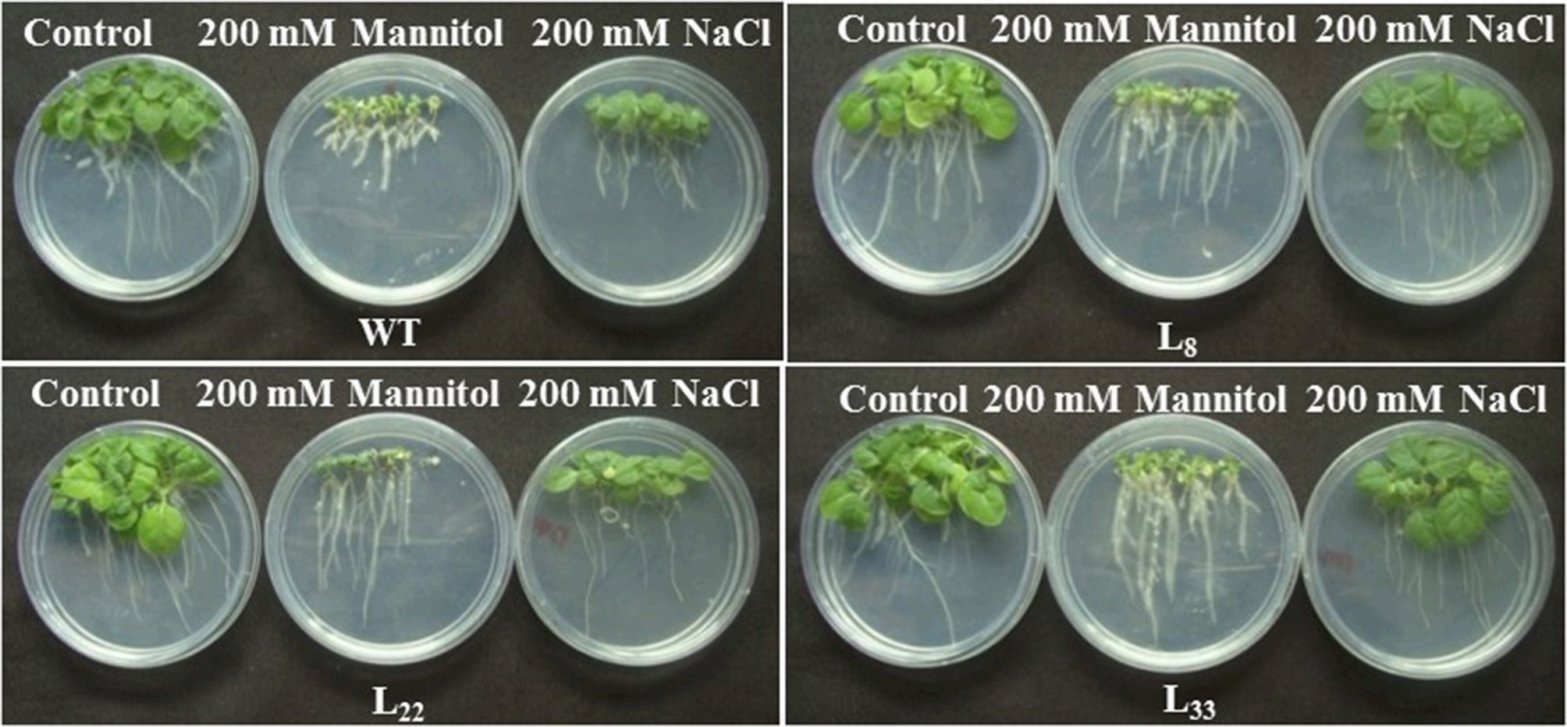
Growth responses of WT and transgenic (L_8_, L_22_, and L_33_) tobacco under drought (200 mM mannitol) and salt (200 mM NaCl) stress conditions for 21 days.

**Figure 4:**
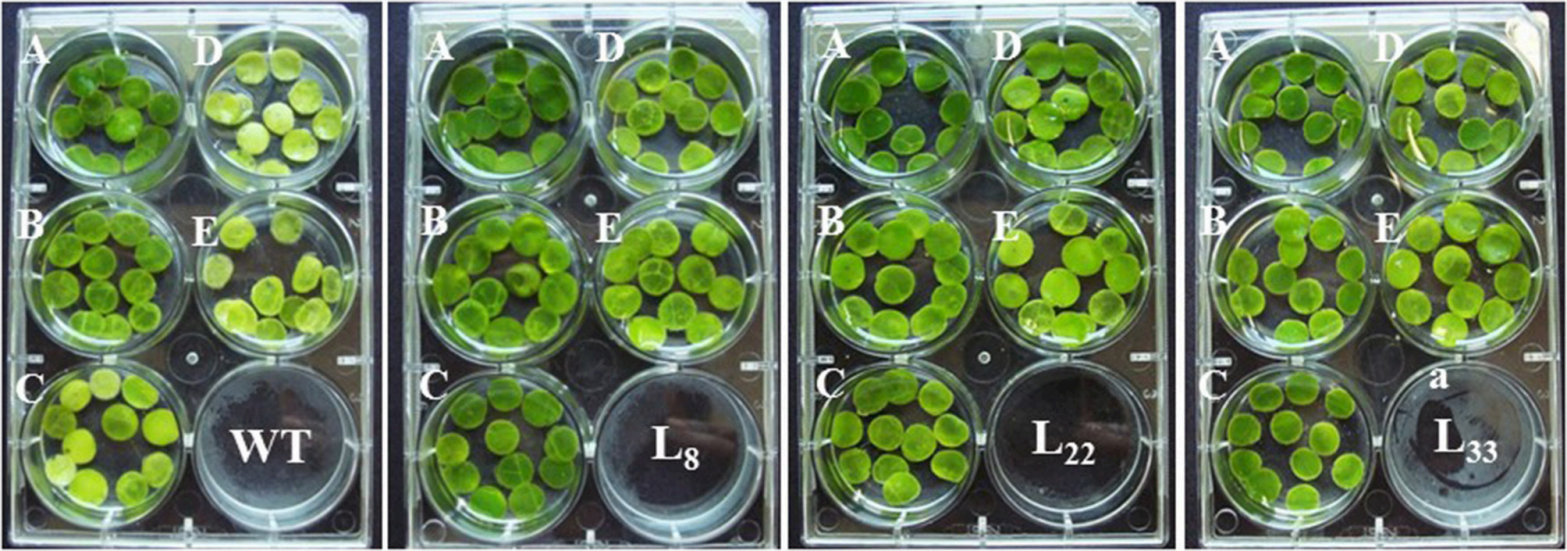
Leaf disc assay of WT and transgenic tobacco under control **(A)** and salt (**B**–100 mM, **C**–150 mM, **D**–200 mM NaCl) and drought (**E**–10% PEG) conditions.

**Figure 5:**
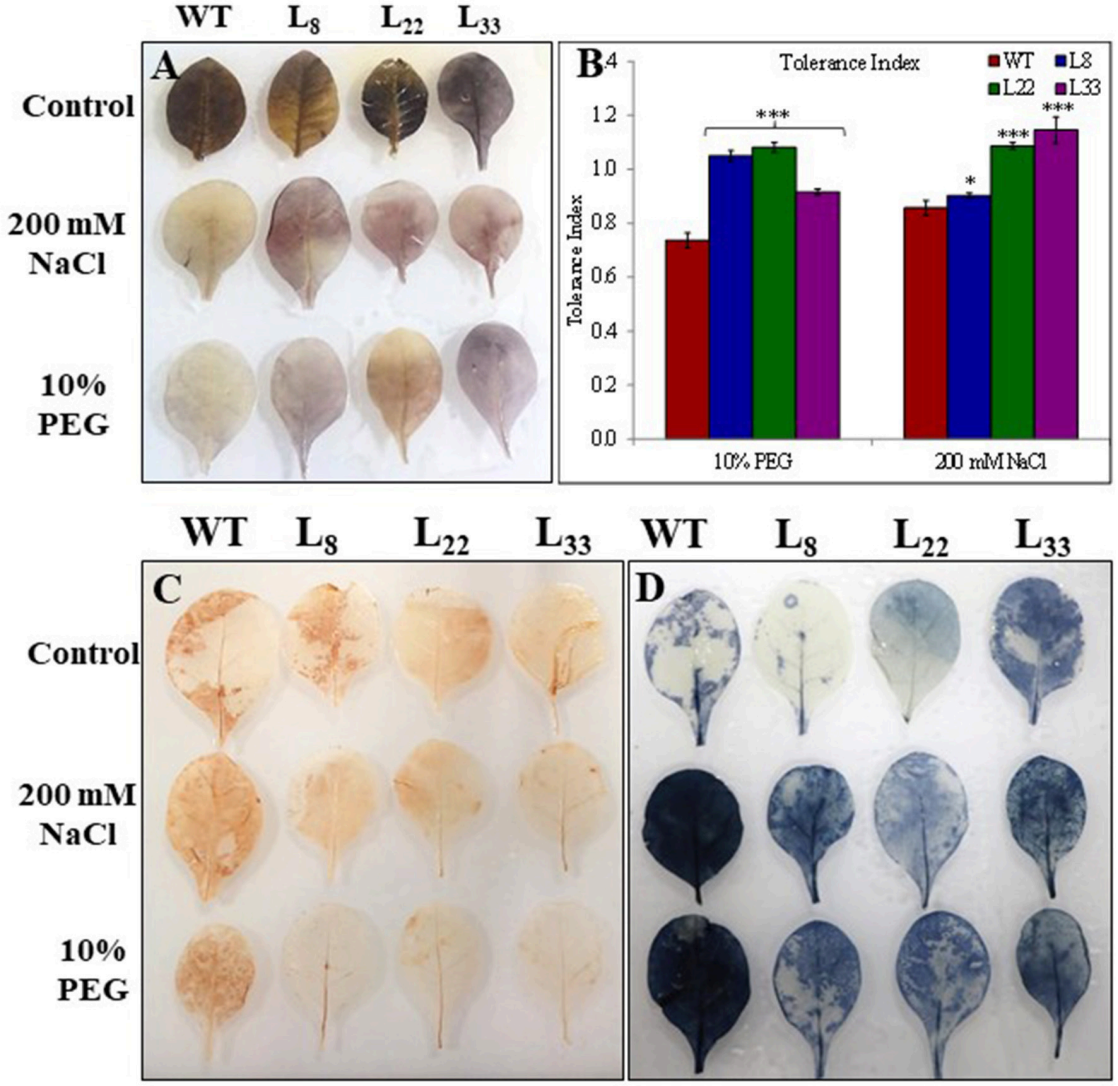
Accumulation of starch in leaves **(A)**, tolerance index **(B)**, and *in vivo* detection of ${\text{O}}_{2}^{-}$ **(C)** and H_2_O_2_ **(D)** in leaves of WT and transgenic tobacco under control and stress (drought and salt) conditions. The ^*^ and ^***^ denote statistical significance in responses of transgenic lines against control at P value ≤0.05 and 0.001 respectively.

The seeds of WT and transgenic lines germinated equally under unstressed conditions (Supplementary Figure 3A) while transgenic lines overexpressing *SbSI*-1 exhibited significantly higher seed germination under stressed conditions (Figure 2A). The transgenic lines exhibited significantly improved growth attributes (Supplementary Figures 3B–F) and grew healthier (Figure 3) than WT under drought and salt stress. In senescence assay, the degree of stress-induced bleaching in leaf disks (Figure 4) and retention of photosynthetic pigments (Figure 2B; Supplementary Figures 4A–C) at the end of stress period showed better endurance in transgenic lines against salt and drought stress. In agreement with these results in TTC assay the transgenic lines exhibited higher cell viability as compared to WT under stress conditions (Figure 2C). The *in vivo* staining of starch showed more accumulation of starch in transgenic lines than WT under stressed conditions indicating better photosynthetic performance by transgenic lines (Figure 5A). Transgenic lines exhibited significantly higher tolerance index (TI) than WT under stressed conditions (Figure 5B). These results clearly evidenced that overexpression of *SbSI*-1 enable transgenic tobacco to perform better and maintain the higher cell viability during the stress period.

### *SbSI*-1 overexpression curtailed ROS induced oxidative damages and improved the physiological health

**Figure 6:**
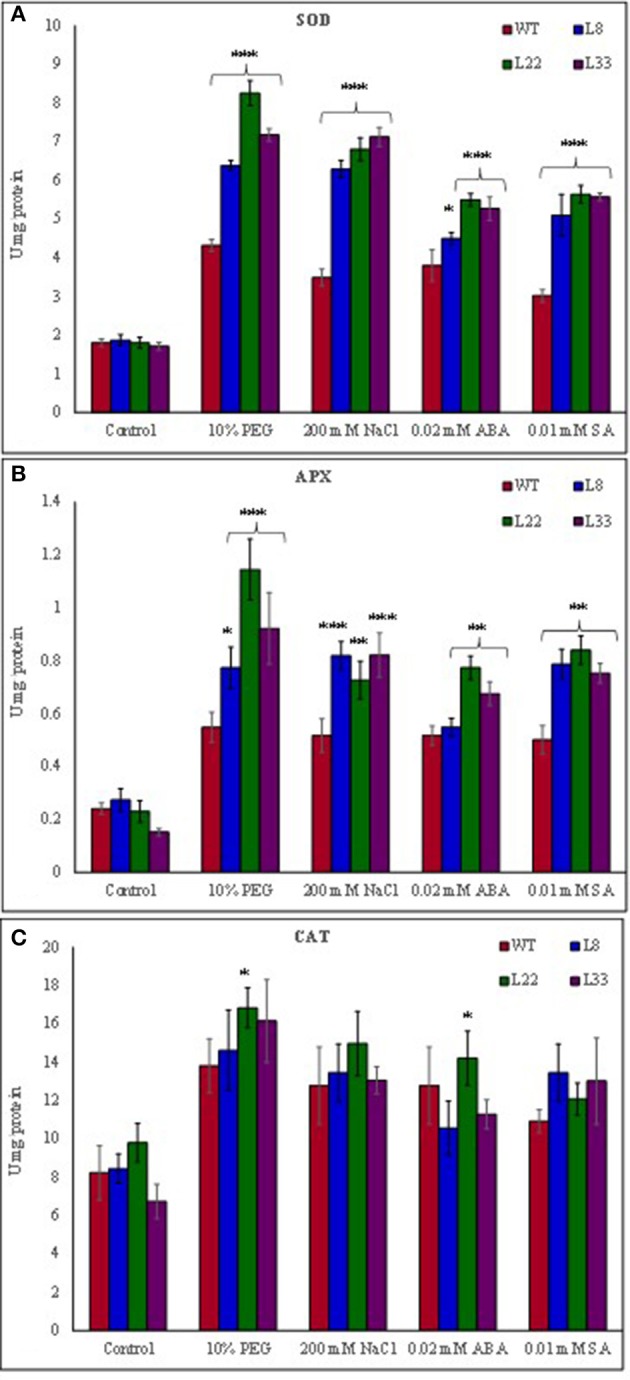
Comparison of activity SOD **(A)**, APX **(B)**, and CAT **(C)** in WT and transgenic tobacco under control and stress (drought and salt) conditions. The ^*^, ^**^, and ^***^ denote statistical significance in responses of transgenic lines against control at P value ≤0.05, 0.01, and 0.001 respectively.

**Figure 7:**
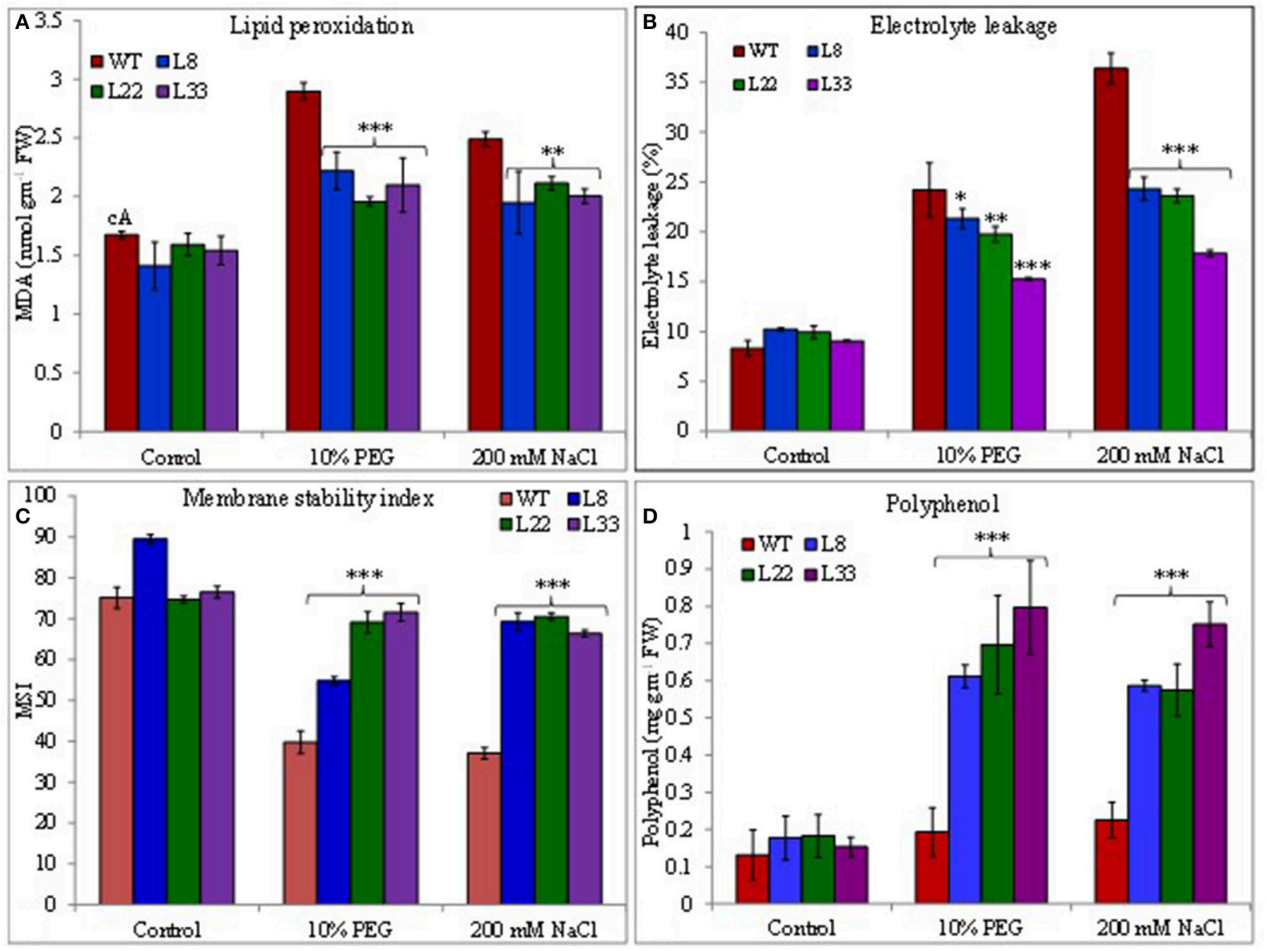
Comparison of **(A)** lipid peroxidation, **(B)** electrolyte leakage, **(C)** membrane stability index, and **(D)** polyphenol contents in WT and transgenic tobacco under control and stress (drought and salt) conditions. The ^*^, ^**^, and ^***^ denote statistical significance in responses of transgenic lines against control at P value ≤0.05, 0.01, and 0.001 respectively.

The abiotic stress leads to the generation of ROS and the plant cope up with excess ROS through its enhanced antioxidant defense system. The activity of SOD, APX and CAT was found higher in both WT and transgenic lines under stress conditions (Figures 6A–C). However, the transgenic lines exhibited significantly higher SOD and APX activities under stress conditions as compared to WT. The transgenic lines exhibited lower accumulation of ${\text{O}}_{2}^{-}$ and H_2_O_2_ than WT tobacco under stressed conditions (Figures 5C,D). In XTT assay the decreased absorbance in transgenic lines as compared to WT under stress conditions confirmed lower generation ${\text{O}}_{2}^{-}$ in transgenic lines overexpressing *SbSI-1* gene (Supplementary Figures 4D). Further the H_2_O_2_ was quantified lower in transgenic lines than WT under stress conditions (Supplementary Figure 4E). These results confirmed that overexpression of *SbSI*-1 alleviates the build-up of ROS (Supplementary Figures 4D,E). In agreement with these results the accumulation of MDA was significantly lower in transgenic lines than WT under stressed conditions (Figure 7A).

The EL an indicator of membrane permeability increased significantly under stressed conditions however it was significantly lower in transgenic lines than WT (Figure 7B). Similarly, the MSI was recorded significantly higher in transgenic lines than WT under stressed conditions (Figure 7C). The polyphenols accumulated significantly higher in leaves of transgenic lines than WT under stressed conditions (Figure 7D). The higher MSI, lower EL, and lower accumulation of MDA in transgenic lines could be considered as proof for curtailment of oxidative damage. The transgenic lines and WT had similar RWC under control conditions while under stressed conditions transgenic lines had significantly higher RWC than WT (Supplementary Figure 4F). These results clearly evident better physiological health of the transgenic tobacco overexpressing *SbSI*-1 than WT under stressed conditions.

Further to study the involvement of *SbSI*-1 in curtailment of ROS induced damages expression pattern of antioxidant genes and transcription factors was examined. The transcript analysis of WT and transgenic lines revealed that expression of the *NtSOD, NtAPX*, and *NtCAT* genes was relatively higher in transgenic lines under different stresses (Supplementary Figures 5A–C). The results indicated higher activity of SOD, APX and CAT in transgenic lines under stressed conditions (Figures 6A–C). Further up-regulation of *NtDREB2* and *NtAP2* in transgenic lines under stress conditions indicated the role of *SbSI*-1 in stress tolerance (Supplementary Figures 5D,E).

### *SbSI*-1 transgenic tobacco had better photosynthetic performance

**Figure 8:**
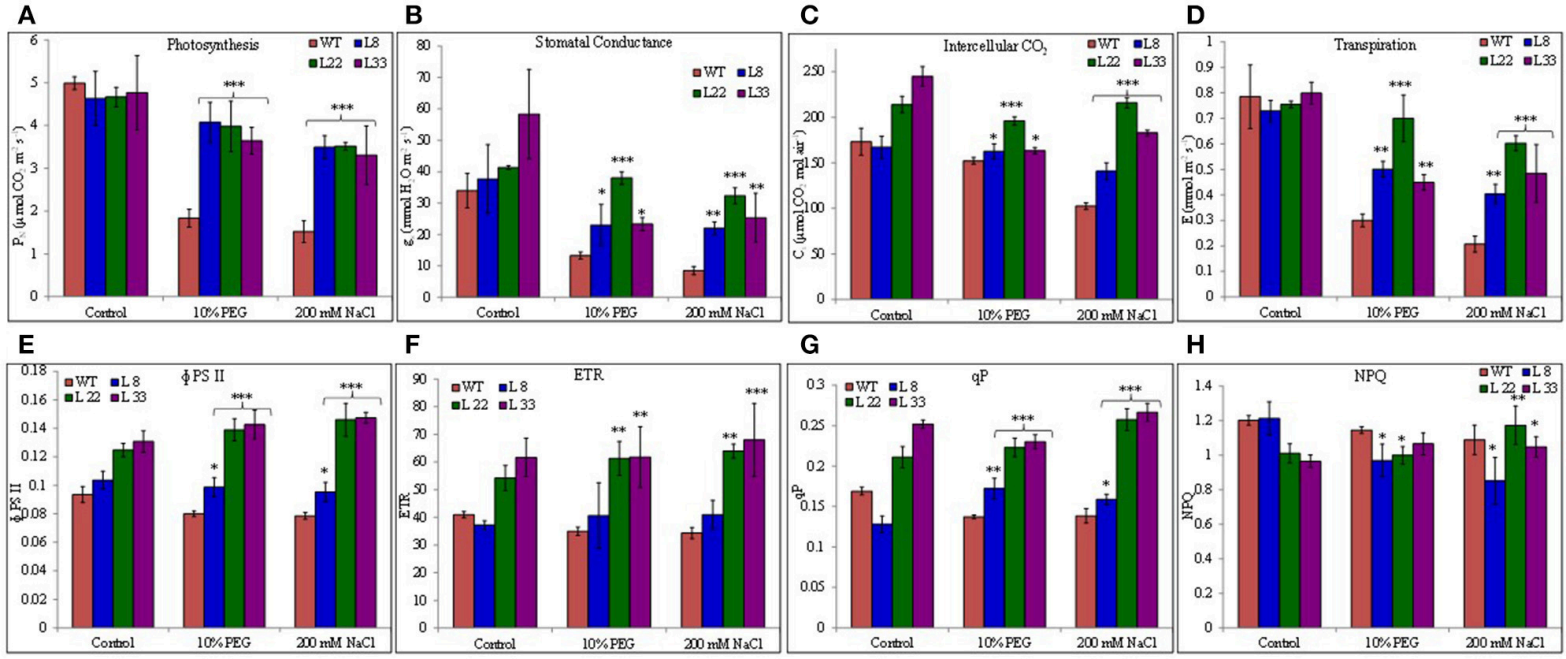
Photosynthetic performances of WT and transgenic tobacco under control and stress (drought and salt) conditions. Comparison of net photosynthesis rate **(A)**, stomatal conductance **(B)**, intercellular CO_2_ concentration **(C)**, transpiration rate **(D)**, PSII operating efficiency **(E)**, electron transport rate **(F)**, photochemical quenching **(G)**, and non-photochemical quenching **(H)** in WT and transgenic tobacco under control and stress (drought and salt) conditions. The ^*^, ^**^, and ^***^ denote statistical significance in responses of transgenic lines against control at P value ≤0.05, 0.01, and 0.001 respectively.

The P_N_, g_s_, and C_i_ decreased in WT and transgenic lines under stressed conditions however the transgenic lines exhibited significantly higher P_N_, g_s_, and C_i_ than WT under drought and salt stress (Figures 8A–C). Similarly the transgenic lines maintained quite higher E than WT (Figure 8D). Transgenic lines exhibited better ΦPSII and higher ETR than WT both under controlled and stressed conditions (Figures 8E,F). In agreement with ΦPSII and ETR, qP was found quite higher in transgenic lines (Figure 8G). The NPQ was comparatively lower in transgenic lines than WT under stressed conditions (Figure 8H) however reduction was non-significant under salt stress. The better P_N_, g_s_, C_i_, E, ΦPSII, ETR, and qP along with the higher accumulation of starch in transgenic lines evidenced better endurance to the drought and salt stress in transgenic tobacco overexpressing *SbSI*-1 gene.

**Figure 9:**
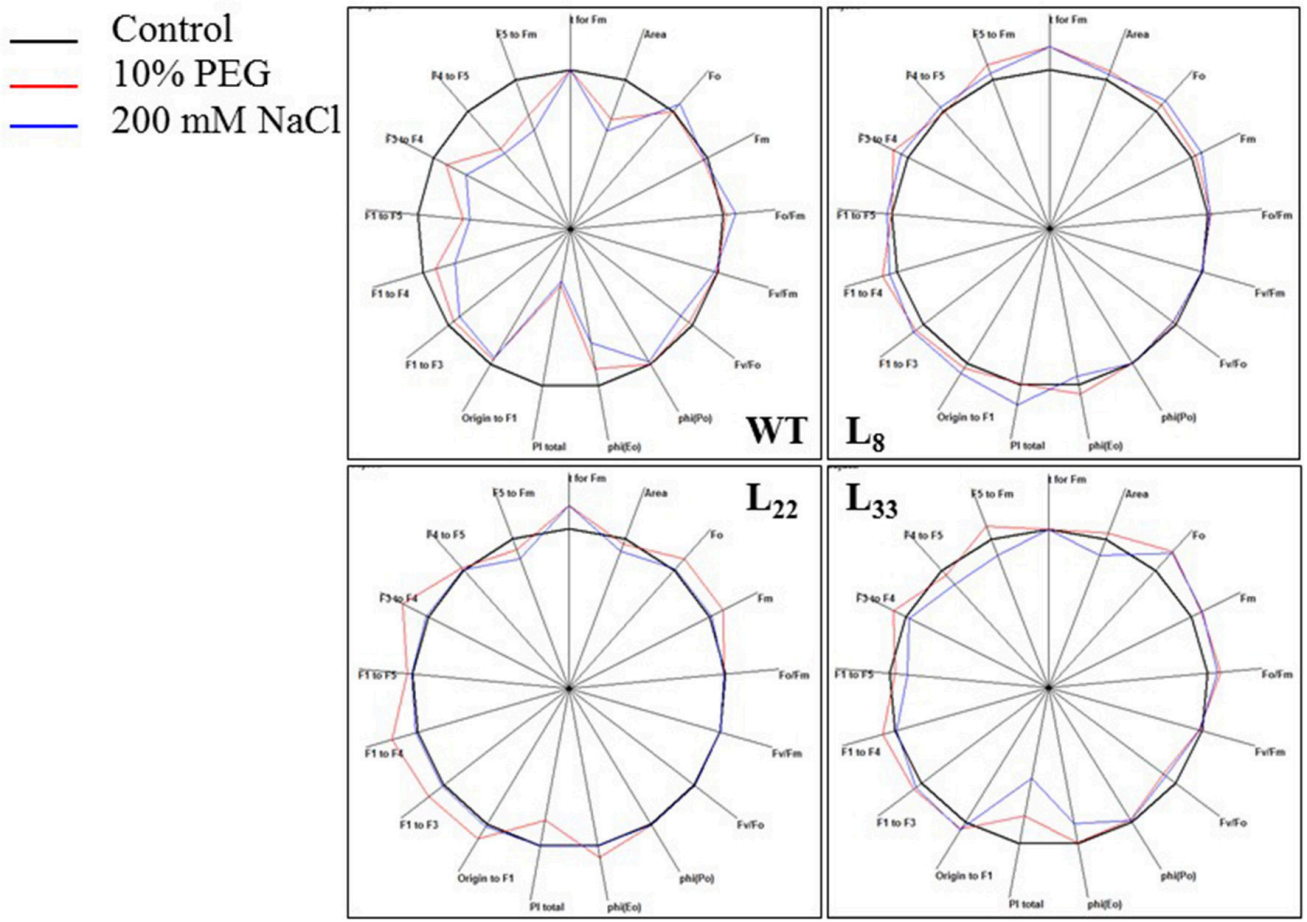
Photosynthetic efficiency of WT and transgenic tobacco. Spider plots of photosynthetic fluxes depicting behavior of Photosystem II in leaves of WT and transgenic (L_8_, L_22_, and L_33_) tobacco under control and stress (drought and salt) conditions.

Chlorophyll a fluorescence transient analysis was performed to corroborate the photosynthetic performance of transgenic lines under stressed conditions (Figure 9). The area over the transient curves (an indicator of plastoquinone pool) increased under drought stress. Under salt stress, it reduced significantly in WT (20.68%) while the reduction was non-significant in transgenic lines. The T_FM_ (time taken to achieve maximum fluorescence) improved comparatively in transgenic lines under drought stress. Under salt stress, T_FM_ decreased, however, lines L_8_ and L_22_ showed improvement. The chlorophyll fluorescence intensity in transgenic lines when all PSII reaction centers are open (F_o_) and closed (F_m_) was comparatively higher than WT under stressed conditions. F_o_/F_m_ (non-photochemical de-excitation) increased under drought and salt stress, however, transgenic lines had relatively lower F_o_/F_m_ than WT. The F_v_/F_m_ (maximum quantum yield of PSII) reduced under stressed conditions and the transgenic lines had relatively higher F_v_/F_m_ than WT. The F_v_/F_o_ (the activity of the water-splitting complex on PSII donor side) reduced under stress conditions while transgenic lines showed relatively better F_v_/F_o_. The TR_o_/ABS (maximum yield of primary photochemistry) and ET_o_/ABS (electron transport flux) reduced in WT under stress conditions while transgenic lines maintained relatively higher TR_o_/ABS and ET_o_/ABS. The PI_Total_ (performance index) decreased significantly in WT under drought (18.22%) and salt (61.02%) stress while it was significantly higher in transgenic lines than WT under stressed conditions.

### *SbSI*-1 overexpression improved osmotic and ion homeostasis

**Figure 10:**
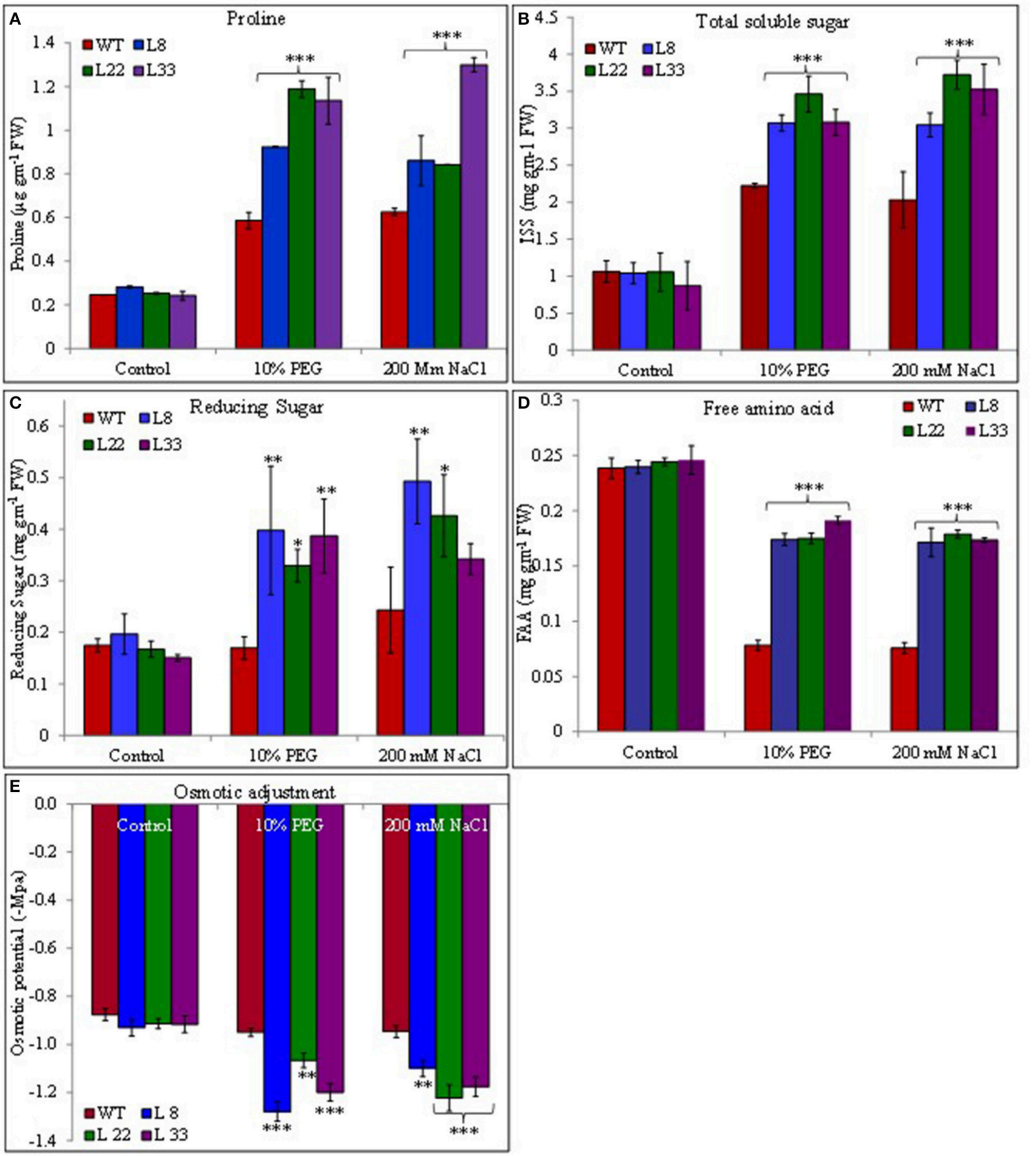
Comparison of proline **(A)**, total soluble sugar **(B)**, reducing sugar **(C)**, free amino acid **(D)** contents, and osmotic potential **(E)** in WT and transgenic tobacco under control and stress (drought and salt) conditions. The ^*^, ^**^, and ^***^ denote statistical significance in responses of transgenic lines against control at P value ≤0.05, 0.01, and 0.001 respectively.

Transgenic and WT plants accumulated proline under stressed conditions however the accumulation was significantly higher in transgenic tobacco than WT plants (Figure 10A). Similarly the soluble sugar and reducing sugar accumulated under stressed conditions and the accumulation was significantly higher in transgenic tobacco than WT (Figures 10B,C). The free amino acids were similar in WT and transgenic tobacco under control conditions. These reduced significantly in WT plants under stress conditions while transgenic tobacco maintained significantly higher accumulation of free amino acids under stress conditions (Figure 10D).

**Figure 11:**
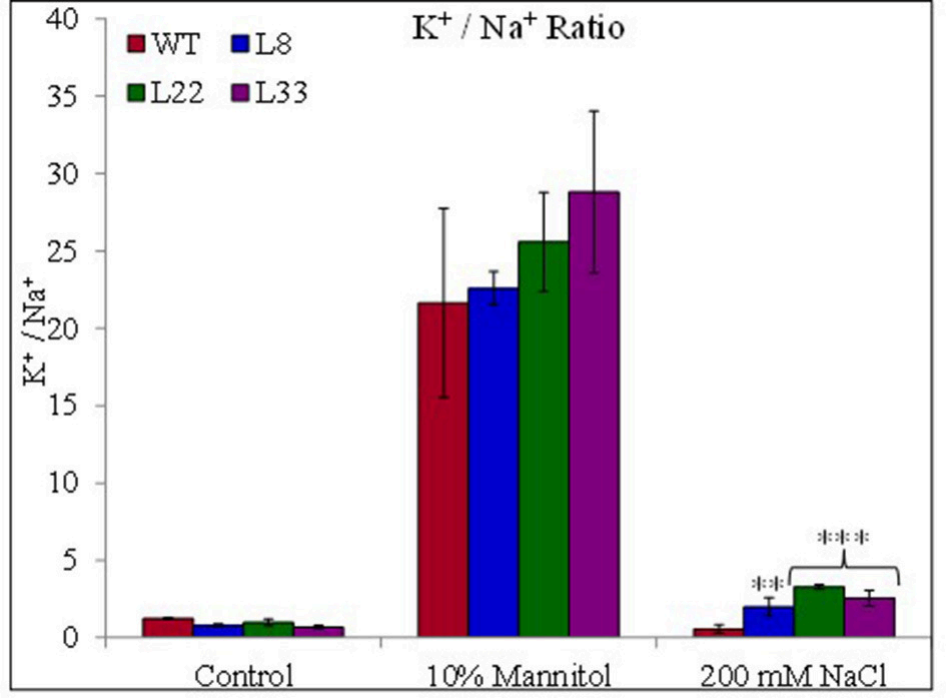
Ionic contents. Comparison of sodium-potassium ratio in WT and transgenic tobacco under control and stress (drought and salt) conditions. The ^**^ and ^***^ denote statistical significance in responses of transgenic lines against control at P value ≤0.01 and 0.001 respectively.

The accumulation of Na^+^ and K^+^ varied in both WT and transgenic tobacco under stress conditions. The transgenic tobacco showed significantly lower accumulation of Na^+^ and higher accumulation of K^+^ compared with that of WT under salt stress (Supplementary Figures 3G,H). Transgenic tobacco significantly accumulated higher K^+^ than WT under drought stress. Similarly, transgenic tobacco had significantly higher K^+^/Na^+^ ratio under stressed conditions (Figure 11). The osmotic potential in WT and transgenic tobacco was recorded similar under control conditions. In agreement with accumulations of organic osmolites and inorganic ions, it increased negatively and the transgenic tobacco showed better osmotic potential than WT (Figure 10E) under stressed conditions.

### Multivariate data analysis of plant responses to drought and salt stress

PCA was carried out both individually for the comparative study of the growth responses, physiological health, osmotic adjustment, and photosynthetic responses; and integrated responses of WT and transgenic tobacco under unstressed and stressed (drought and salt) conditions. Individually growth responses, physiological health, osmotic adjustment and photosynthetic responses PCA revealed 79.47, 83.76, 82.87, and 78.34% variations, respectively (Supplementary Figures 6A–D). The WT and transgenic tobacco showed comparable response under control and stressed conditions as revealed by clustering of transgenic tobacco plants under particular stress in the individual bi-plot analysis. Clustering of transgenic tobacco plants under particular stress showed comparable responses to avoid the harmful effect of stresses. The integrated PCA revealed 68.36% variations (Supplementary Figures 6A–D). Both the individual and integrated PCA showed the possible correlations of plant response to different variables and stress condition (Supplementary Figures 6E). Overall, the PCA showed a statistical difference among growth responses, physiological health, osmotic adjustment and photosynthetic responses of WT and transgenic tobacco under control and stress conditions.

## Discussion

Earlier the heterologous expression of *SbSI*-1 conferred the desiccation and salt tolerance in *E. coli* (Yadav et al., 2012). In this study, we elucidated *in planta* role of *SbSI*-1 gene in drought and salt stress tolerance using transgenic tobacco. The *in vivo* subcellular localization confirmed SbSI-1 as a nuclear protein. The protein showed DNA binding property (Uguru et al., 2005; Stead et al., 2006). Nuclear localization indicated it as an essential element of abiotic stress responses (Yadav et al., 2014) like other stress-associated proteins (Tamirisa et al., 2014; Lakra et al., 2015; Wang et al., 2015; Yang et al., 2015). The transgenic tobacco overexpressing *SbSI*-1 grew normal under control conditions and showed better seed germination, plant growth and biomass as compared to WT plants under stress conditions (Yadav et al., 2014; Udawat et al., 2016). A lower degree of chlorophyll bleaching in senescence assay and better retention of photosynthetic pigments under different stresses showed better tolerance of transgenic tobacco (Lakra et al., 2015). The higher cell viability in the transgenic tobacco compared with WT tobacco under stress conditions indicated reduced oxidative damage and better survival of transgenic lines. The higher cell viability helped better retention of photosynthetic pigments and the leaf disks exhibited enhanced greenness in leaf disc senescence assay. The better growth performance of transgenic tobacco overexpressing *SbSI*-1 compared with that of WT plants under stress conditions revealed the role of *SbSI*-1 in overcoming the deleterious effects of stress.

The ROS influence various aspects of plant biology (Foyer and Noctor, 2016; Sewelam et al., 2016) and different stresses cause increased accumulation of ROS (Mittler, 2002; Miller et al., 2008). Excess accumulation of ROS cause membrane lipid peroxidation, DNA damage, denaturation of protein/enzymes and pigment breakdown (Bose et al., 2014) resulting in oxidative damages. The antioxidant enzyme system help plants to scavenge the excess ROS (Jebara et al., 2005) and minimize the oxidative damages. Under stress conditions the higher activities of SOD, APX and CAT in transgenic lines indicated the enhanced scavenging of ROS thus lowering the ROS accumulation (Huang et al., 2011). The MDA concentration is an indicator of membrane damage and lower accumulation of MDA in transgenic tobacco under stress treatments clearly show reduced damage to the cell membrane in transgenic tobacco than WT tobacco. Further higher MSI and lower EL in transgenic tobacco under stress conditions clearly indicated membrane stability. In accordance with these results, the *in vivo* localization of ${\text{O}}_{2}^{-}$ and H_2_O_2_ showed lower accumulation of the ROS in transgenic lines under stress conditions. Further XTT assay and H_2_O_2_ quantification confirmed lower accumulation of the ROS. The proline performs multiple antioxidant functions (Bose et al., 2014) and the polyphenols have super ability to scavenge the ROS. Increased accumulation of proline and polyphenol help the transgenic tobacco to reduce the build-up of ROS. These results clearly evidenced the lower accumulation of ROS and a significant level of protection in transgenic tobacco overexpressing *SbSI*-1 under stressed conditions.

Salt and drought stress disrupt the metabolic balance of the cell and cause injuries to cell membrane, nucleic acid and photosynthetic pigments (Mittler, 2002). The RWC, EL, MSI, OP, TI, proline, and MDA concentration are indicators of physiological health of a plant. The transgenic tobacco overexpressing *SbSI*-1 showed significantly higher MSI and this supports the reduced EL in transgenic tobacco under stressed conditions. The EL and MSI may be correlated with several physiological functions in plants. Further higher content of MDA in WT as compared to transgenic tobacco under stressed conditions support membrane stabilization in transgenic tobacco. The transgenic tobacco retained higher RWC than WT under stressed conditions and similarly the TI of transgenic tobacco was determined higher. The reduced EL, higher RWC and MSI along with higher TI evident the role *SbSI*-1 in membrane protection under stress condition and counteract the physiological drought.

Photosynthetic characteristics unveil the physiological performance of the plants under stressed conditions. The lower carbon assimilation in WT plants under stressed conditions suggested an accumulation of electrons at PSI acceptor side resulting ROS generation and PSI photo-inhibition. The transgenic tobacco had higher P_N_ than WT tobacco probably due to higher g_s_ and C_i_. Higher E in transgenic tobacco helped to maintain the leaf temperature and reduced the chances of thermal damage to the photosynthetic apparatus. ΦPSII indicate photosynthetic performances of plants and it was significantly higher in transgenic tobacco. Similarly, transgenic tobacco showed higher ETR and qP than WT under stressed conditions. The higher electron transport through PSI and PSII systems improves the functionality of photosynthetic apparatus through improving ATP and NADPH production, which in turn support CO_2_ fixation in dark reactions. The lower NPQ in transgenic tobacco under stressed conditions indicated reduced heat loss. Higher ΦPSII, ETR and qP and lower NPQ along with higher g_s_ and C_i_ accounted for higher P_N_ in transgenic tobacco under stressed conditions. Further higher starch accumulation in transgenic tobacco under stressed conditions reflected the higher carbon assimilation (Yang et al., 2014).

The different stresses affect the photosynthetic performance of the plants by influencing the leaf's ability to channel solar energy through the photochemical pathways. Chlorophyll fluorescence analysis is widely employed in plant stress analysis (Papageorgiou and Govindjee, 2004; Kalaji et al., 2011; Salvatori et al., 2014). The higher plastoquinone pool in transgenic tobacco than WT under stressed conditions indicate better electron transfer from the reaction centers to the quinone pool hence better photosynthesis. Similarly, higher electron transport flux in transgenic tobacco accounted for improved electron transport rate under stressed conditions. The maximum quantum yield of PSII (F_v_/F_m_) is an indicator of PSII efficiency and reflects photoinhibition of PSII (Oukarroum et al., 2013; Yang et al., 2014). Lower F_v_/F_m_ in WT under stressed conditions indicated photo-oxidative damage to PSII. Higher chlorophyll fluorescence and F_v_/F_m_ in transgenic tobacco evident the maintenance of photosynthetic efficiencies under stressed conditions while higher chlorophyll fluorescence and reduced F_v_/F_m_ in WT plants indicated the structural damage to PSII reaction center (Salvatori et al., 2014). The non-photochemical de-excitation was significantly higher in WT than transgenic tobacco under stressed conditions. The stressed environment caused a decrease in the activity of water-splitting complex and it was more prominent in WT than transgenic tobacco. The higher performance index in transgenic tobacco than WT under stressed conditions indicated the role of *SbSI*-1 in maintaining the photosynthetic efficiency.

The transgenic tobacco showed higher accumulation of sugar than WT under stressed conditions. This indicates the improved photosynthetic efficiencies of transgenic tobacco and the increase sugar content help in osmotic adjustment. The stress-stimulated polyphenol accumulation participates in defense against ROS (Ksouri et al., 2007). Transgenic tobacco showed higher accumulation of proline and free amino acids than WT under stressed conditions. The role of proline is well established as an osmoprotectant under stressed conditions. Free amino acids serve as raw material for protein synthesis and also act as regulatory and signaling molecules (Ali et al., 2015). These compatible solutes perform multiple functions under stressed environment and their higher accumulations indicated beneficial physiological processes in transgenic tobacco overexpressing *SbSI*-1. In addition to conventional roles the osmolites act as low-molecular-weight chaperones, stabilize the PSII system, protect the protein/enzyme structure, maintain the membrane integrity and scavenge ROS (Chen et al., 2007). The K^+^/Na^+^ ratio, an essential requirement for the optimum metabolic functioning of plant cells is an important ion homeostasis parameter under stressed conditions. The improved K^+^/Na^+^ ratio indicated better stress tolerance of transgenic tobacco compared with that of WT. The reduced leakage of K^+^, ROS accumulation, and higher transpiration rate might be the probable reasons for maintenance of higher concentration of Na^+^ and K^+^ in the transgenic lines as compared to WT under mannitol stress (Demidchik et al., 2014; Sun et al., 2015). Salt and drought tolerant plants are well equipped to adjust the tissue water potential more negative than that of the soil water potential of the habitat (Khan et al., 2000). In the present study, transgenic tobacco overexpressing *SbSI*-1 had more negative osmotic potential than WT plants under stress conditions, indicating better adaptability of transgenic tobacco (Tamirisa et al., 2014). Further, the higher accumulation of compatible solutes and the large K^+^/Na^+^ ratio can be correlated with better osmotic adjustment in transgenic tobacco (Pardo and Quintero, 2002; Udawat et al., 2016).

The level of ROS-induced damages in transgenic tobacco under stress conditions revealed the role of *SbSI*-1 gene directly (as a non-enzymatic antioxidant) or indirectly (through up-regulation of genes encoding antioxidant enzymes) in alleviating the ROS build-up. The ROS-scavenging activity cut down the build-up of ROS and this is considered as an important component of salt-tolerance mechanisms (Zhu, 2001). The drought, salt, ABA, and SA treatments induced the transcript of *NtSOD, NtCAT, NtAPX, NtDREB2*, and *NtAP2*. The variations in the level of transcript of these genes might be due to positional effect of transgene (Al-Shawi et al., 1990), effect of transgene and differences in the conversion of amount of transcribed mRNA into protein (Kanobe et al., 2013), level of epigenetic modifications (Vilperte et al., 2016) and differential sensitivity of lines to a particular stress. The up-regulations of transcripts of these genes suggest that the *SbSI*-1 help plants to detoxify/scavenge the ROS under stress conditions. The up-regulation of DREB2 and AP2 under stress conditions indicated the role of *SbSI*-1 in abiotic stress tolerance mechanism. A similar pattern of transcript expression of antioxidant genes and the transcription factors with ABA and stress conditions in transgenic tobacco suggested that the *SbSI*-1 act in an ABA-dependent manner. Also a similar pattern of transcript expression of antioxidant genes and the transcription factors with SA and stress treatments confirmed the role of *SbSI*-1 in stress tolerance mechanism.

The *in planta* validation clearly established the role of *SbSI*-1 in drought and salt stress tolerance through ion homeostasis, osmotic homeostasis and low build-up of ROS. Under stress conditions, possibly the expression of *SbSI*-1 induce/up-regulate the genes for (i) synthesis of compatible solutes for osmotic homeostasis, (ii) transporter/ion channel for redox homeostasis and (iii) synthesis of proteinaceous (enzymatic) and non-enzymatic antioxidant for mitigation of excess ROS accumulation. The ROS and redox homeostasis help plant to maintain physiological integrity while osmotic homeostasis makes water available for different physiological processes. The better osmotic, redox and ROS homeostasis in transgenic tobacco overexpressing *SbSI*-1 under stressed conditions indicate curtailment of oxidative damages and beneficial physiological processes.

## Conclusion

*In planta* validation of *SbSI*-1 ascertained that the overexpression of *SbSI*-1 conferred drought and salt tolerance in transgenic tobacco. The overexpression of *SbSI*-1 improved the physiological health of transgenic tobacco under stressed conditions by curtailing oxidative damage and maintaining photosynthetic efficiency, however, additional studies required to elucidate the exact mode of action. Taken together, the findings suggest that *SbSI*-1 enhance drought and salt stress tolerance through multiple physiological pathways and may be considered as a potential candidate gene for improving stress tolerance in crop plants.

## Author contributions

Conceptualized, conceived, and designed the experiments: MR and BJ. Performed the experiment: JK, PU, MH, and AD. Data analysis: JK, PU, and MR. Manuscript preparation: MR.

### Conflict of interest statement

The authors declare that the research was conducted in the absence of any commercial or financial relationships that could be construed as a potential conflict of interest.

## Supplementary material

Supplementary Figure 1 Schematic maps of gene constructs and GUS staining of transgenic tobacco: *pSITE-4CA* vector **(A)**, RFP:*SbSI*-1 gene construct **(B)**, Plant expression gene construct pCAMBIA2301-*SbSI*-1 **(C)**, and DNA protein binding assay **(D)** and Histochemical GUS staining of WT and selected *SbSI*-1 transgenic tobacco **(E)**.

Supplementary Figure 2 Details of putative transgenic lines: Histochemical GUS-staining of putative lines **(A)**, Molecular confirmation of putative transgenic lines by PCR amplification of *uidA* gene, and *SbSI-1* gene **(B)** and semi-quantitative RT-PCR in *SbSI*-1 and *uid*A PCR positive transgenic tobacco lines **(C)**. Lane M is ladder, WT is wild type, PC-positive control and 1–42 are line numbers.

Supplementary Figure 3 Growth parameters and ion contents: Comparison of seed germination **(A)**, whole plant growth **(B)**, shoot and root growth **(C,D)**, fresh and dry weight **(E,F)**, potassium and sodium contents **(G,H)** in WT and transgenic tobacco grown under control and stress (200 mM mannitol and 200 mM NaCl) conditions for 21 days. The ^*^, ^**^, and ^***^ denote statistical significance in responses of transgenic lines against control at P value ≤0.05, 0.01, and 0.001 respectively.

Supplementary Figure 4 Pigment contents and Physiological health: Comparison of chlorophyll a **(A)**, chlorophyll b **(B)**, carotenoids **(C)** contents, superoxide radical accumulation **(D)**, H_2_O_2_ accumulation **(E)**, and relative water contents **(F)** in WT and transgenic tobacco under control and stress (PEG and NaCl) conditions. The ^*^, ^**^, and ^***^ denote statistical significance in responses of transgenic lines against control at P value ≤0.05, 0.01, and 0.001 respectively.

Supplementary Figure 5 Expression analysis of *NtSOD* **(A)**, *NtAPX* **(B)**, *NtCAT* **(C)**, *NtDREB2* **(D)** and *NtAP2* **(E)** in WT and transgenic tobacco under control and different stress (10% PEG, 200 mM NaCl, 20 μM ABA and 10 μM Salicylic acid) condition. The ^*^, ^**^, and ^***^ denote statistical significance in responses of transgenic lines against control at P value ≤0.05, 0.01, and 0.001 respectively.

Supplementary Figure 6 Principal component analysis of individual growth responses **(A)**, physiological health **(B)**, osmotic adjustment, **(C)** photosynthetic responses **(D)**, and integrated responses **(E)** of WT and transgenic tobacco under control and stress (drought and salt) conditions.

Supplementary Table 1 Primer sets used for the confirmation and transcript analysis of transgenic lines.

## References

[B1] Able A. J. Guest D. I. Sutherland M. W. (1998). Use of a new Tetrazolium-based assay to study the production of superoxide radicals by Tobacco cell cultures challenged with avirulent zoospores of phytophthora parasitica var nicotianae. Plant Physiol. 117, 491–499. 10.1104/pp.117.2.491 9625702 PMC34969

[B2] Ali M. S. Kim K. W. Dhakal R. Choi D. Baek K. H. (2015). Accumulation of high contents of free amino acids in the leaves of *Nicotiana benthamiana* by the co-suppression of *NbClpC1* and *NbClpC2* genes. Plant Cell Rep. 34, 355–365. 10.1007/s00299-014-1714-4 25433858

[B3] Al-Shawi R. A. Y. A. Kinnaird J. Burke J. Bishop J. O. (1990). Expression of a foreign gene in a line of transgenic mice is modulated by a chromosomal position effect. Mol. Cell. Biol. 10, 1192–1198. 10.1128/mcb.10.3.1192 2304463 PMC360995

[B4] Amtmann A. Bohnert H. J. Bressan R. A. (2005). Abiotic stress and plant genome evolution. search for new models. Plant Physiol. 138, 127–130. 10.1104/pp.105.059972 15888685 PMC1104168

[B5] Beyer W. F. Fridovich I. (1987). Assaying for superoxide dismutase activity: some large consequences of minor changes in conditions. Anal. Biochem. 161, 559–566. 10.1016/0003-2697(87)90489-1 3034103

[B6] Bose J. Rodrigo-Moreno A. Shabala S. (2014). ROS homeostasis in halophytes in the context of salinity stress tolerance. J. Exp. Bot. 65, 1241–1257. 10.1093/jxb/ert430 24368505

[B7] Bradford M. M. (1976). A rapid and sensitive method for the quantitation of microgram quantities of protein utilizing the principle of protein-dye binding. Anal. Biochem. 72, 248–254. 10.1016/0003-2697(76)90527-3 942051

[B8] Chamovitz D. Sandmann G. Hirschberg J. (1993). Molecular and biochemical characterization of herbicide-resistant mutants of cyanobacteria reveals that phytoene desaturation is a rate-limiting step in carotenoid biosynthesis. J. Biol. Chem. 268, 17348–17353. 8349618

[B9] Chandler S. F. Dodds J. H. (1983). The effect of phosphate, nitrogen and sucrose on the production of phenolics and solasodine in callus cultures of *Solanum laciniatum*. Plant Cell Rep. 2, 205–208. 10.1007/BF00270105 24258053

[B10] Chen Z. Cuin T. A. Zhou M. Twomey A. Naidu B. P. Shabala S. (2007). Compatible solute accumulation and stress-mitigating effects in barley genotypes contrasting in their salt tolerance. J. Exp. Bot. 58, 4245–4255. 10.1093/jxb/erm284 18182428

[B11] Cheng Y. Zi-shan Z. Hui-yuan G. Xing-li F. Mei-jun L. Xiang-dong L. (2014). The mechanism by which NaCl treatment alleviates PSI photoinhibition under chilling-light treatment. J. Photoch. Photobio. B 140, 286–291. 10.1016/j.jphotobiol.2014.08.012 25194527

[B12] Demidchik V. Straltsova D. Medvedev S. S. Pozhvanov G. A. Sokolik A. Yurin V. (2014). Stress-induced electrolyte leakage: the role of K+-permeable channels and involvement in programmed cell death and metabolic adjustment. J. Exp. Bot. 65, 1259–1270. 10.1093/jxb/eru004 24520019

[B13] Dubois M. Gilles K. A. Hamilton J. K. Rebers P. Smith F. (1956). Colorimetric method for determination of sugars and related substances. Anal. Chem. 28, 350–356. 10.1021/ac60111a017

[B14] Foyer C. H. Noctor G. (2016). Stress-triggered redox signalling: what's in pROSpect? Plant Cell Environ. 39, 951–964. 10.1111/pce.12621 26264148

[B15] Hasegawa P. M. Bressan R. A. Zhu J. K. Bohnert H. J. (2000). Plant cellular and molecular responses to high salinity. Annu. Rev. Plant Biol. 51, 463–499. 10.1146/annurev.arplant.51.1.463 15012199

[B16] He Z. Wang Z. Y. Li J. Zhu Q. Lamb C. Ronald P.. (2000). Perception of brassinosteroids by the extracellular domain of the receptor kinase BRI1. Science 288, 2360–2363. 10.1126/science.288.5475.2360 10875920

[B17] Hema R. Vemanna R. S. Sreeramulu S. Reddy C. P. Senthil-Kumar M. Udayakumar M. (2014). Stable expression of mtlD gene imparts multiple stress tolerance in finger millet. PLoS ONE 9:e99110. 10.1371/journal.pone.0099110 24922513 PMC4055669

[B18] Hodges D. M. DeLong J. M. Forney C. F. Prange R. K. (1999). Improving the thiobarbituric acid-reactive-substances assay for estimating lipid peroxidation in plant tissues containing anthocyanin and other interfering compounds. Planta 20, 604–611. 10.1007/s004250050524 28456836

[B19] Horsch R. B. Fry J. E. Hoffmann N. L. Eichholtz D. Rogers S. A. Fraley R. T. (1985). A simple and general method for transferring genes into plants. Science 227, 1229–1231. 10.1126/science.227.4691.1229 17757866

[B20] Huang X. S. Luo T. Fu X. Z. Fan Q. J. Liu J. H. (2011). Cloning and molecular characterization of a mitogen-activated protein kinase gene from *Poncirus trifoliata* whose ectopic expression confers dehydration/drought tolerance in transgenic tobacco. J. Exp. Bot. 62, 5191–5206. 10.1093/jxb/err229 21778184 PMC3193021

[B21] Inskeep W. P. Bloom P. R. (1985). Extinction coefficients of chlorophyll a and b in N, N-dimethylformamide and 80% acetone. Plant Physiol. 77, 483–485. 10.1104/pp.77.2.483 16664080 PMC1064541

[B22] Jebara S. Jebara M. Limam F. Aouani M. E. (2005). Changes in ascorbate peroxidase, catalase, guaiacol peroxidase and superoxide dismutase activities in common bean (*Phaseolus vulgaris*) nodules under salt stress. J. Plant Physiol. 162, 929–936. 10.1016/j.jplph.2004.10.005 16146319

[B23] Jefferson R. A. (1987). Assaying chimeric genes in plants: the GUS gene fusion system. Plant Mol. Biol. Rep. 5, 387–405. 10.1007/BF02667740

[B24] Jha B. Agarwal P. K. Reddy P. S. Lal S. Sopory S. K. Reddy M. K. (2009). Identification of salt-induced genes from *Salicornia brachiata*, an extreme halophyte through expressed sequence tags analysis. Genes Genet. Syst. 84, 111–120. 10.1266/ggs.84.111 19556705

[B25] Kalaji H. M. Bosa K. Kościelniak J. Żuk-Gołaszewska K. (2011). Effects of salt stress on photosystem II efficiency and CO_2_ assimilation of two Syrian barley landraces. Environ. Exp. Bot. 73, 64–72. 10.1016/j.envexpbot.2010.10.009

[B26] Kanobe M. N. Rodermel S. R. Bailey T. Scott P. (2013). Changes in endogenous gene transcript and protein levels in maize plants expressing the soybean ferritin transgene. Front. Plant Sci. 4:196. 10.3389/fpls.2013.00196 23785377 PMC3682644

[B27] Katschnig D. Bliek T. Rozema J. Schat H. (2015). Constitutive high-level SOS1 expression and absence of HKT1; 1 expression in the salt-accumulating halophyte Salicornia dolichostachya. Plant Sci. 234, 144–154. 10.1016/j.plantsci.2015.02.011 25804817

[B28] Khan M. A. Ungar I. A. Showalter A. M. (2000). Effects of salinity on growth, water relations and ion accumulation of the subtropical perennial halophyte, *Atriplex griffithii* var. stocksii. Ann. Bot. 85, 225–232. 10.1006/anbo.1999.1022

[B29] Ksouri R. Megdiche W. Debez A. Falleh H. Grignon C. Abdelly C. (2007). Salinity effects on polyphenol content and antioxidant activities in leaves of the halophyte *Cakile maritima*. Plant Physiol. Biochem. 45, 244–249. 10.1016/j.plaphy.2007.02.001 17408958

[B30] Lakra N. Nutan K. K. Das P. Anwar K. Singla-Pareek S. L. Pareek A. (2015). A nuclear-localized histone-gene binding protein from rice (*OsHBP1b*) functions in salinity and drought stress tolerance by maintaining chlorophyll content and improving the antioxidant machinery. J. Plant Physiol. 176, 36–46. 10.1016/j.jplph.2014.11.005 25543954

[B31] Livak K. J. Schmittgen T. D. (2001). Analysis of relative gene expression data using real-time quantitative PCR and the 2^−Δ*ΔCT*^ method. Methods 25, 402–408. 10.1006/meth.2001.1262 11846609

[B32] Lv S. Jiang P. Chen X. Fan P. Wang X. Li Y. (2012). Multiple compartmentalization of sodium conferred salt tolerance in Salicornia europaea. Plant Physiol. Biochem. 51, 47–52. 10.1016/j.plaphy.2011.10.015 22153239

[B33] Miller G. L. (1959). Use of dinitrosalicylic acid reagent for determination of reducing sugar. Anal. Chem. 31, 426–428. 10.1021/ac60147a030

[B34] Miller G. Shulaev V. Mittler R. (2008). Reactive oxygen signalling and abiotic stress. Physiol. Plant 133, 481–489. 10.1111/j.1399-3054.2008.01090.x 18346071

[B35] Mittler R. (2002). Oxidative stress, antioxidants and stress tolerance. Trends Plant Sci. 7, 405–410. 10.1016/S1360-1385(02)02312-9 12234732

[B36] Miyagawa Y. Tamoi M. Shigeoka S. (2000). Evaluation of the defense system in chloroplasts to photooxidative stress caused by paraquat using transgenic tobacco plants expressing catalase from Escherichia coli. Plant Cell Physiol. 41, 311–320. 10.1093/pcp/41.3.311 10805594

[B37] Murashige T. Skoog F. (1962). A revised medium for rapid growth and bio assays with tobacco tissue cultures. Physiol. Plant. 15, 473–497. 10.1111/j.1399-3054.1962.tb08052.x

[B38] Nakano Y. Asada K. (1981). Hydrogen peroxide is scavenged by ascorbate-specific peroxidase in spinach chloroplasts. Plant Cell Physiol. 22, 867–880

[B39] Nath M. Yadav S. Sahoo R. K. Passricha N. Tuteja R. Tuteja N. (2016). PDH45 transgenic rice maintain cell viability through lower accumulation of Na^+^, ROS and calcium homeostasis in roots under salinity stress. J. Plant Physiol. 191, 1–11. 10.1016/j.jplph.2015.11.008 26687010

[B40] Oukarroum A. Goltsev V. Strasser R. J. (2013). Temperature effects on pea plants probed by simultaneous measurements of the kinetics of prompt fluorescence, delayed fluorescence and modulated 820 nm reflection. PLoS ONE 8:59433. 10.1371/journal.pone.0059433 23527194 PMC3602342

[B41] Papageorgiou G. C. Govindjee (2004). Chlorophyll a fluorescence: a signature of photosynthesis, in Advances in Photosynthesis and Respiration, Vol. 19, ed Govindjee (Dordrecht: Springer).

[B42] Pardo J. M. Quintero F. J. (2002). Plants and sodium ions: keeping company with the enemy. Genome Biol. 3, reviews1017.1–reviews1017.4. 10.1186/gb-2002-3-6-reviews1017 12093381 PMC139373

[B43] Patterson B. D. Payne L. A. Chen Y. Z. Graham D. (1984). An inhibitor of catalase induced by cold in chilling-sensitive plants. Plant Physiol. 76, 1014–1018. 10.1104/pp.76.4.1014 16663941 PMC1064426

[B44] Ramegowda V. Senthil-Kumar M. Nataraja K. N. Reddy M. K. Mysore K. S. Udayakumar M. (2012). Expression of a finger millet transcription factor, *EcNAC1*, in tobacco confers abiotic stress-tolerance. PLoS ONE 7:40397. 10.1371/journal.pone.0040397 22808152 PMC3394802

[B45] Rathore M. S. Paliwal N. Anand K. V. Agarwal P. K. (2015). Somatic embryogenesis and *in vitro* plantlet regeneration in *Salicornia brachiata* Roxb. Plant Cell Tiss. Org. Cult. 120, 355–360. 10.1007/s11240-014-0571-8

[B46] Reddy P. C. O. Sairanganayakulu G. Thippeswamy M. Reddy P. S. Reddy M. K. Sudhakar C. (2008). Identification of stress-induced genes from the drought tolerant semi-arid legume crop horsegram (*Macrotyloma uniflorum* (Lam.) Verdc.) through analysis of subtracted expressed sequence tags. Plant Sci. 175, 372–384. 10.1016/j.plantsci.2008.05.012

[B47] Ringel C. Siebert S. Wienhaus O. (2003). Photometric determination of proline in quartz microplates: remarks on specificity. Anal. Biochem. 313, 167–169. 10.1016/S0003-2697(02)00565-1 12576073

[B48] Sairam R. K. (1994). Effects of homobrassinolide application on plant metabolism and grain yield under irrigated and moisture-stress conditions of two wheat varieties. Plant Growth Regul. 14, 173–181. 10.1007/BF00025220

[B49] Salvatori E. Fusaro L. Gottardini E. Pollastrini M. Goltsev V. Strasser R. J.. (2014). Plant stress analysis: application of prompt, delayed chlorophyl fluorescence and 820 nm modulated reflectance. insights from independent experiments. Plant Physiol. Biochem. 85, 105–113. 10.1016/j.plaphy.2014.11.002 25463266

[B50] Schopfer P. Plachy C. Frahry G. (2001). Release of reactive oxygen intermediates (superoxide radicals, hydrogen peroxide, and hydroxyl radicals) and peroxidase in germinating radish seeds controlled by light, gibberellin, and abscisic acid. Plant Physiol. 125, 1591–1602. 10.1104/pp.125.4.1591 11299341 PMC88817

[B51] Sewelam N. Kazan K. Schenk P. M. (2016). Global plant stress signaling: reactive oxygen species at the cross-road. Front. Plant Sci. 7:187. 10.3389/fpls.2016.00187 26941757 PMC4763064

[B52] Shukla P. S. Gupta K. Agarwal P. Jha B. Agarwal P. K. (2015). Overexpression of a novel *SbMYB15* from *Salicornia brachiata* confers salinity and dehydration tolerance by reduced oxidative damage and improved photosynthesis in transgenic tobacco. Planta 242, 1291–1308. 10.1007/s00425-015-2366-5 26202734

[B53] Singh D. Yadav N. S. Tiwari V. Agarwal P. Jha B. (2016a). A SNARE-like superfamily protein *SbSLSP* from the halophyte *Salicornia brachiata* confers salt and drought tolerance by maintaining membrane stability, K^+^/Na^+^ ratio, and antioxidant machinery. Front. Plant Sci. 7:737. 10.3389/fpls.2016.00737 27313584 PMC4889606

[B54] Singh V. K. Mishra A. Haque I. Jha B. (2016b). A novel transcription factor-like gene *SbSDR1* acts as a molecular switch and confers salt and osmotic endurance to transgenic tobacco. Sci. Rep. 6:31686. 10.1038/srep31686 27550641 PMC4994045

[B55] Srivastava V. K. Raikwar S. Tuteja R. Tuteja N. (2016). Ectopic expression of phloem motor protein pea forisome PsSEO-F1 enhances salinity stress tolerance in tobacco. Plant Cell Rep. 35, 1021–1041. 10.1007/s00299-016-1935-9 26825595

[B56] Stead J. A. Keen J. N. McDowall K. J. (2006). The identification of nucleic acid-interacting proteins using a simple proteomics-based approach that directly incorporates the electrophoretic mobility shift assay. Mol. Cell. Proteomics 5, 1697–1702. 10.1074/mcp.T600027-MCP200 16845145

[B57] Strasser B. J. Strasser R. J. (1995). Measuring fast fluorescence transients to address environmental questions: the JIP-test, in Photosynthesis: From Light to Biosphere, ed Mathis P. (Dordrecht: Kluwer Academic Publishers), 977–980.

[B58] Sugano N. Tanaka T. Yamamoto E. Nishi A. (1975). Behaviour of phenylalanine ammonia-lyase in carrot cells in suspension cultures. Photochemistry 14, 2435–2436. 10.1016/0031-9422(75)80359-1

[B59] Sun Y. Kong X. Li C. Liu Y. Ding Z. (2015). Potassium retention under salt stress is associated with natural variation in salinity tolerance among arabidopsis accessions. PloS ONE 10:e0124032. 10.1371/journal.pone.0124032 25993093 PMC4438003

[B60] Sutherland M. W. Learmonth B. A. (1997). The tetrazolium dyes MTS and XTT provide new quantitative assays for superoxide and superoxide dismutase. Free Radical Res. 27, 283–289. 10.3109/10715769709065766 9350432

[B61] Tamirisa S. Vudem D. R. Khareedu V. R. (2014). Overexpression of pigeonpea stress-induced cold and drought regulatory gene (CcCDR) confers drought, salt, and cold tolerance in Arabidopsis. J. Exp. Bot. 65, 4769–4781. 10.1093/jxb/eru224 24868035 PMC4144763

[B62] Tiwari V. Chaturvedi A. K. Mishra A. Jha B. (2013). The transcriptional regulatory mechanism of the peroxisomal ascorbate peroxidase (*pAPX)* gene cloned from an extreme halophyte *Salicornia brachiata*. Plant Cell Physiol. 55, 1774–1871. 10.1093/pcp/pct172 24285755

[B63] Tiwari V. Patel M. K. Chaturvedi A. K. Mishra A. Jha B. (2016). Functional characterization of the tau class glutathione-s-transferases gene (*sbgstu*) promoter of *Salicornia brachiata* under salinity and osmotic stress. PLoS ONE 11:e0148494. 10.1371/journal.pone.0148494 26885663 PMC4757536

[B64] Töpfer R. Matzeit V. Gronenborn B. Schell J. Steinbiss H. H. (1987). A set of plant expression vectors for transcriptional and translational fusions. Nucleic Acids Res. 15:5890. 10.1093/nar/15.14.5890 3615207 PMC306034

[B65] Towill L. E. Mazur P. (1975). Studies on the reduction of 2, 3, 5-triphenyltetrazolium chloride as a viability assay for plant tissue cultures. Can. J. Bot. 53, 1097–1102. 10.1139/b75-129

[B66] Tuteja N. Sahoo R. K. Garg B. Tuteja R. (2013). *OsSUV3* dual helicase functions in salinity stress tolerance by maintaining photosynthesis and antioxidant machinery in rice (*Oryzasativa*, L. cv. *IR64*). Plant J. 76, 115–127. 10.1111/tpj.12277 23808500

[B67] Udawat P. Jha R. K. Sinha D. Mishra A. Jha B. (2016). Overexpression of a cytosolic abiotic stress responsive universal stress protein (*SbUSP*) mitigates salt and osmotic stress in transgenic tobacco plants. Front. Plant Sci. 7:518. 10.3389/fpls.2016.00518 27148338 PMC4838607

[B68] Uguru G. C. Stephens K. E. Stead J. A. Towle J. E. Baumberg S. McDowall K. J. (2005). Transcriptional activation of the pathway-specific regulator of the actinorhodin biosynthetic genes in *Streptomyces coelicolor*. Mol. Microbiol. 58, 131–150. 10.1111/j.1365-2958.2005.04817.x 16164554

[B69] Vilperte V. Agapito-Tenfen S. Z. Wikmark O. G. Nodari R. O. (2016). Levels of DNA methylation and transcript accumulation in leaves of transgenic maize varieties. Environ. Sci. Eur. 28, 29. 10.1186/s12302-016-0097-2 27942424 PMC5120055

[B70] Vinocur B. Altman A. (2005). Recent advances in engineering plant tolerance to abiotic stress: achievements and limitations. Curr. Opin. Biotechnol. 16, 123–132. 10.1016/j.copbio.2005.02.001 15831376

[B71] Walhout A. J. Temple G. F. Brasch M. A. Hartley J. L. Lorson M. A. van den Heuvel S. (2000). GATEWAY recombinational cloning: application to the cloning of large numbers of open reading frames or ORFeomes. Method Enzymol. 328, 575–592. 10.1016/S0076-6879(00)28419-X 11075367

[B72] Wang F. Tong W. Zhu H. Kong W. Peng R. Liu Q.. (2015). A novel Cys2/His2 zinc finger protein gene from sweet potato *IbZFP1*, is involved in salt and drought tolerance in transgenic *Arabidopsis*. Planta 243, 783–797. 10.1007/s00425-015-2443-9 26691387

[B73] Wang Z. L. Li P. H. Fredricksen M. Gong Z. Z. Kim C. S. Zhang C. (2004). Expressed sequence tags from *Thellungiella halophila*, a new model to study plant salt-tolerance. Plant Sci. 166, 609–616. 10.1016/j.plantsci.2003.10.030

[B74] Yadav N. S. Rashmi D. Singh D. Agarwal P. K. Jha B. (2012). A novel salt-inducible gene SbSI-1 from *Salicornia brachiata* confers salt and desiccation tolerance in *E. coli*. Mol. Biol. Rep. 39, 1943–1948. 10.1007/s11033-011-0941-9 21655957

[B75] Yadav N. S. Singh V. K. Singh D. Jha B. (2014). A novel gene SbSI-2 encoding nuclear protein from a halophyte confers abiotic stress tolerance in *E. coli* and tobacco. PloS ONE 9:101926. 10.1371/journal.pone.0101926 24999628 PMC4084957

[B76] Yang C. Zhang Z. S. Gao H. Y. Fan X. L. Liu M. J. Li X. D. (2014). The mechanism by which NaCl treatment alleviates PSI photo inhibition under chilling-light treatment. J. Photochem. Photobiol. B. Biol. 140, 286–291. 10.1016/j.jphotobiol.2014.08.012 25194527

[B77] Yang X. Wang X. Ji L. Yi Z. Fu C. Ran J. (2015). Over-expression of a *Miscanthus lutarioriparius* NAC gene MlNAC5 confers enhanced drought and cold tolerance in *Arabidopsis*. Plant Cell 34, 943–958. 10.1007/s00299-015-1756-2 25666276

[B78] Zhu J. K. (2001). Cell signaling under salt, water and cold stresses. Curr. Opin. Plant Biol. 4, 401–406. 10.1016/S1369-5266(00)00192-8 11597497

