# Supplementary figures and images for: Overexpression of SbSI-1, A Nuclear Protein from Salicornia brachiata Confers Drought and Salt Stress Tolerance and Maintains Photosynthetic Efficiency in Transgenic Tobacco
Source: Front Plant Sci. 2017 Jul 13;8:1215. doi: 10.3389/fpls.2017.01215 (PMC5508026; doi:10.3389/fpls.2017.01215)

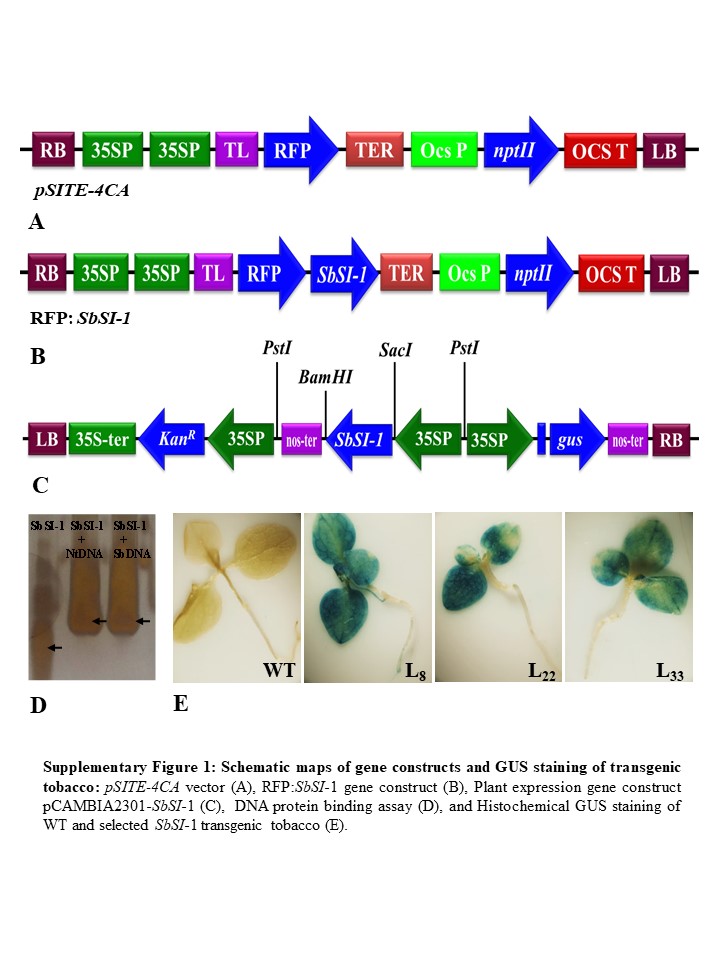

Supplement: Supplementary Figure 1 — Schematic maps of gene constructs and GUS staining of transgenic tobacco: pSITE-4CA vector (A), RFP:SbSI-1 gene construct (B), Plant expression gene construct pCAMBIA2301-SbSI-1 (C), and DNA protein binding assay (D) and Histochemical GUS staining of WT and selected SbSI-1 transgenic tobacco (E). [file Image1.jpg]

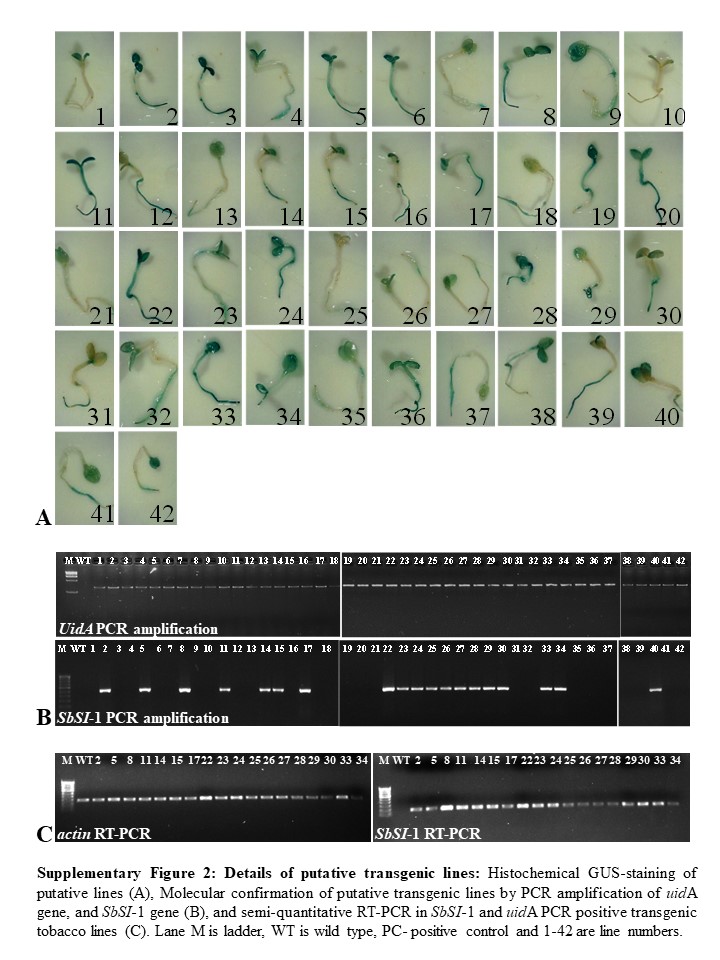

Supplement: Supplementary Figure 2 — Details of putative transgenic lines: Histochemical GUS-staining of putative lines (A), Molecular confirmation of putative transgenic lines by PCR amplification of uidA gene, and SbSI-1 gene (B) and semi-quantitative RT-PCR in SbSI-1 and uidA PCR positive transgenic tobacco lines (C). Lane M is ladder, WT is wild type, PC-positive control and 1–42 are line numbers. [file Image2.jpg]

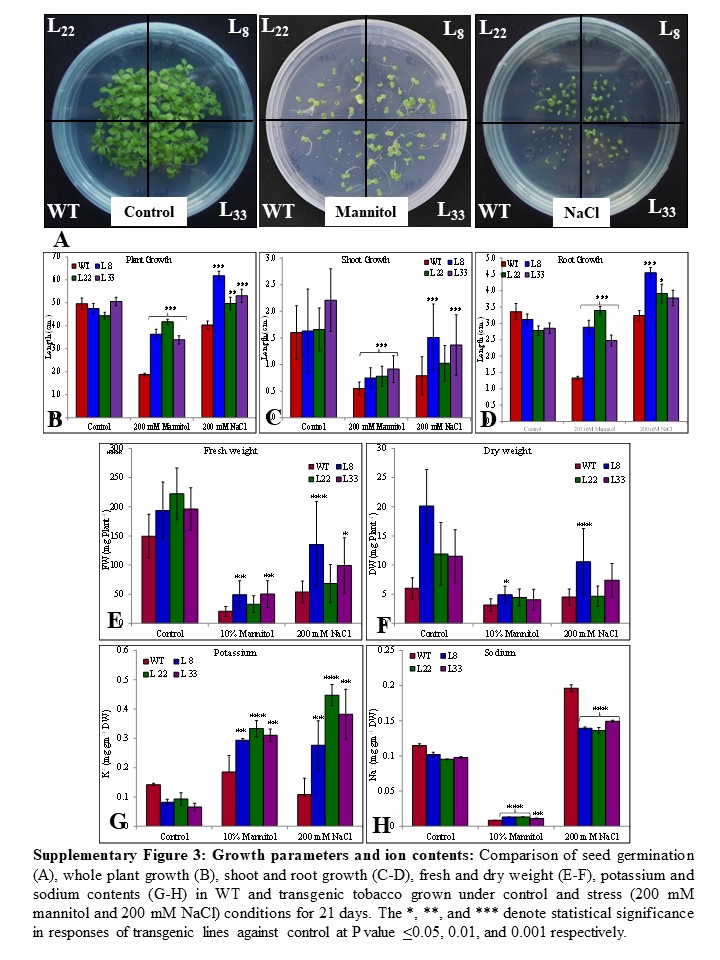

Supplement: Supplementary Figure 3 — Growth parameters and ion contents: Comparison of seed germination (A), whole plant growth (B), shoot and root growth (C,D), fresh and dry weight (E,F), potassium and sodium contents (G,H) in WT and transgenic tobacco grown under control and stress (200 mM mannitol and 200 mM NaCl) conditions for 21 days. The *, **, and *** denote statistical significance in responses of transgenic lines against control at P value ≤0.05, 0.01, and 0.001 respectively. [file Image3.jpg]

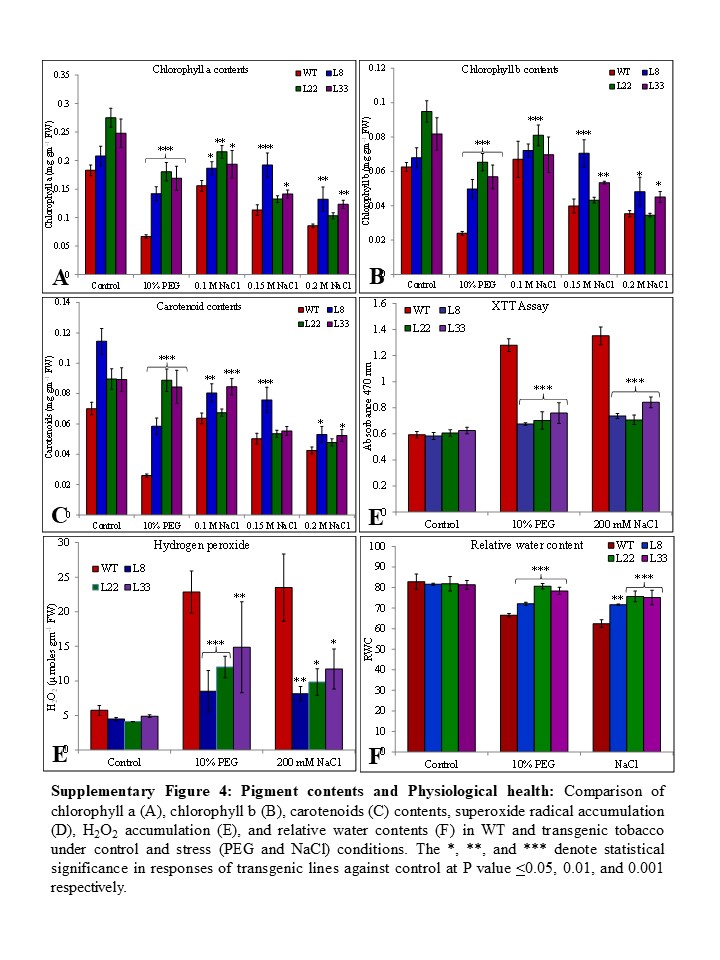

Supplement: Supplementary Figure 4 — Pigment contents and Physiological health: Comparison of chlorophyll a (A), chlorophyll b (B), carotenoids (C) contents, superoxide radical accumulation (D), H2O2 accumulation (E), and relative water contents (F) in WT and transgenic tobacco under control and stress (PEG and NaCl) conditions. The *, **, and *** denote statistical significance in responses of transgenic lines against control at P value ≤0.05, 0.01, and 0.001 respectively. [file Image4.jpg]

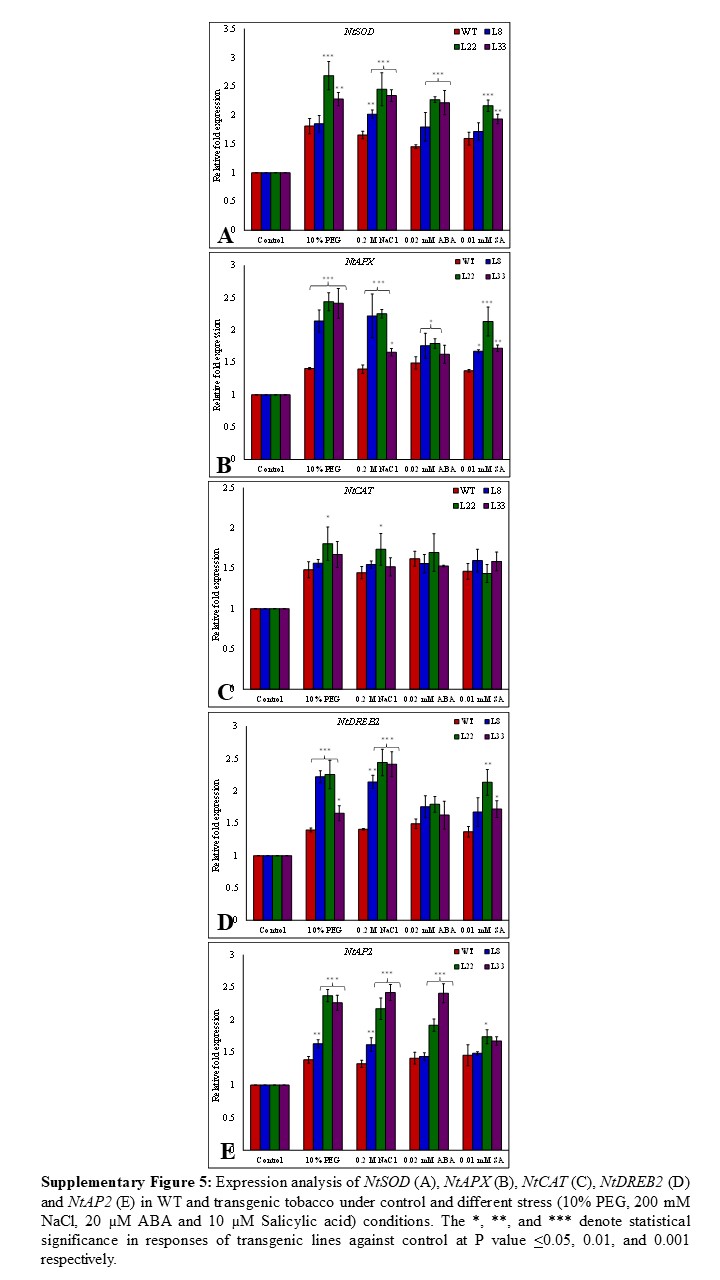

Supplement: Supplementary Figure 5 — Expression analysis of NtSOD (A), NtAPX (B), NtCAT (C), NtDREB2 (D) and NtAP2 (E) in WT and transgenic tobacco under control and different stress (10% PEG, 200 mM NaCl, 20 μM ABA and 10 μM Salicylic acid) condition. The *, **, and *** denote statistical significance in responses of transgenic lines against control at P value ≤0.05, 0.01, and 0.001 respectively. [file Image5.jpg]

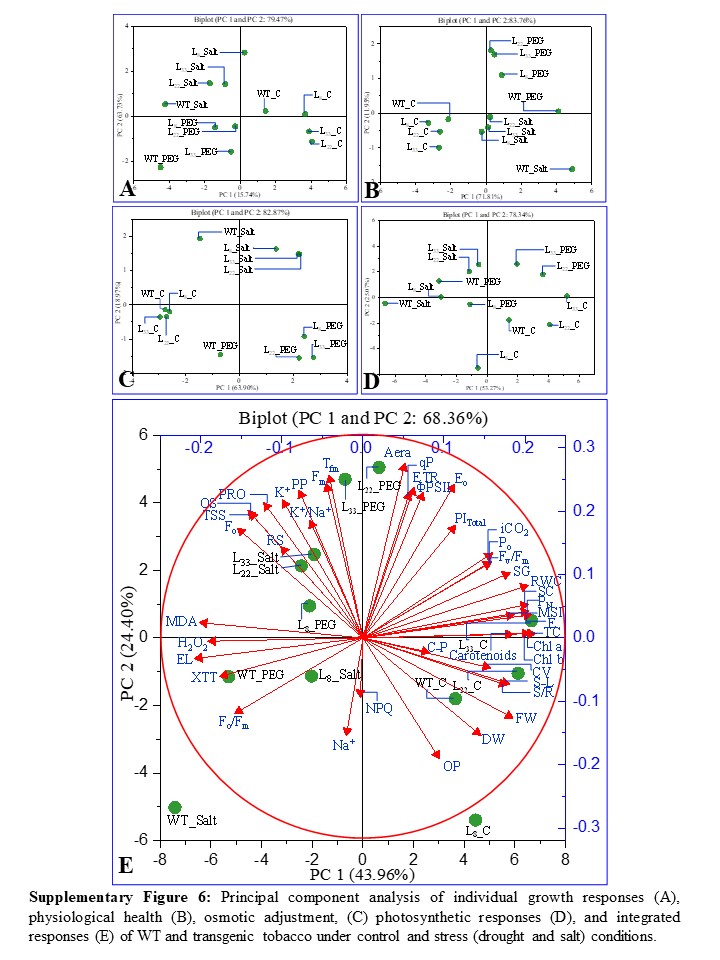

Supplement: Supplementary Figure 6 — Principal component analysis of individual growth responses (A), physiological health (B), osmotic adjustment, (C) photosynthetic responses (D), and integrated responses (E) of WT and transgenic tobacco under control and stress (drought and salt) conditions. [file Image6.jpg]
