# Supplementary material for: Overexpression of SbSI-1, A Nuclear Protein from Salicornia brachiata Confers Drought and Salt Stress Tolerance and Maintains Photosynthetic Efficiency in Transgenic Tobacco
Source: Front Plant Sci. 2017 Jul 13;8:1215. doi: 10.3389/fpls.2017.01215 (PMC5508026; doi:10.3389/fpls.2017.01215)
Supplement: Supplementary Table 1 — Primer sets used for the confirmation and transcript analysis of transgenic lines. [file Table1.DOCX]

**Supplementary table 1:** Primer sets used for the confirmation and transcript analysis of transgenic lines.

| S. No. | Gene / Purpose | Primer sequence (5’→3’) | PCR conditions |
| --- | --- | --- | --- |
| 1. | *SbSI-1* | F: ATGCCTAATAAACATATCATGG | 94 ºC, 5 min; 32 Cycles: 94 ºC, 30 s; 58 ºC, 40 s; 72 ºC, 50s; 72 ºC, 10 min; 4 ºC hold |
|  |  | R: TTAACGGTTCCCTTGTTTC |  |
| 2. | *uid*A | F: GATCGCGAAAACTGTGGAAT | 94 ºC, 5min; 32 Cycles: 94 ºC, 45s; 58 ºC, 45s; 72 ºC, 1 min; Extension: 72 ºC, 7 min; 4 ºC hold |
|  |  | R: TGAGCGTCGCAGAACATTAC |  |
| 3. | *Actin* | F: CGTTTGGATCTTGCTGGTCGT | 94 °C for 1 min; 32 cycles: 94 °C for 40 s; 55 °C for 60 s and 72 °C for 60 s; final extension at 72 °C for 10 min; 4 ºC hold |
|  |  | R: CAGCAATGCCAGGGAACATAG |  |
| 4. | *NtAPX* | F: CAAATGTAAGAGGAAACTCAGAGGA | 94 °C for 1 min; 40 cycles: 94 °C for 10 s; 60 °C for 10 s and 72 °C for 15 s; final extension at 72 °C for 10 min; 4 ºC hold |
|  |  | R: CAGCCTTGAGCCTCATGGTACCG |  |
| 5. | *NtSOD* | F: AGCTACATGACGCCATTTCC |  |
|  |  | R: CCCTGTAAAGCAGCACCTTC |  |
| 6. | *NtCAT* | AGGTACCGCTCATTCACACC |  |
|  |  | AAGCAAGCTTTTGACCCAGA |  |
| 7. | *NtAP2* | F: AAGGGCGAGGAAGAACAAAT |  |
|  |  | F: GTGGCTCTGGAA AGTTGA |  |
| 8. | *NtDREB2* | F: GCCGACGCTAAGGATA TTCA |  |
|  |  | F:TGCAAAACAGAGCTTCCTCA |  |
| 9. | Localization | *SbSI-1*LF 5’-CACCATGCCTAATAAACATATCATGG-3’ | 94 ºC, 5min; 35 Cycles: 94 ºC, 30s; 55 ºC, 30s; 72 ºC, 30s; Extension: 72 ºC, 10 min; 4 ºC hold |
|  |  | *SbSI-1*LR 5’-TTAACGGTTCCCTTGTTTC-3’ |  |
